# Time-space-resolved origami hierarchical electronics for ultrasensitive detection of physical and chemical stimuli

**DOI:** 10.1038/s41467-019-09070-8

**Published:** 2019-03-08

**Authors:** Min Zhang, Jiaxing Jeccy Sun, Muhammad Khatib, Zi-Yang Lin, Zi-Han Chen, Walaa Saliba, A’laa Gharra, Yehu David Horev, Viki Kloper, Yana Milyutin, Tan-Phat Huynh, Simon Brandon, Guoyue Shi, Hossam Haick

**Affiliations:** 1 Department of Chemical Engineering and Russell Berrie Nanotechnology Institute, Technion - Israel Institute of Technology, 320003 Haifa, Israel; 2 School of Chemistry and Molecular Engineering, Shanghai Key Laboratory for Urban Ecological Processes and Eco-Restoration, East China Normal University, 500 Dongchuan Road, 200241 Shanghai, China; 3 Laboratory of Physical Chemistry, Faculty of Science and Engineering, Åbo Akademi University, Porthaninkatu 3-5, FI-20500 Turku, Finland

## Abstract

Recent years have witnessed thriving progress of flexible and portable electronics, with very high demand for cost-effective and tailor-made multifunctional devices. Here, we report on an ingenious origami hierarchical sensor array (OHSA) written with a conductive ink. Thanks to origami as a controllable hierarchical framework for loading ink material, we have demonstrated that OHSA possesses unique time-space-resolved, high-discriminative pattern recognition (TSR-HDPR) features, qualifying it as a smart sensing device for simultaneous sensing and distinguishing of complex physical and chemical stimuli, including temperature, relative humidity, light and volatile organic compounds (VOCs). Of special importance, OSHA has shown very high sensitivity in differentiating between structural isomers and chiral enantiomers of VOCs – opening a door for wide variety of unique opportunities in several length scales.

## Abstract (web summary)

Developing portable, disposable and cost-effective electronics for multifunctional sensing is desirable. Here, the authors present origami-based hierarchical electronics with time-space-resolved high-discriminative pattern recognition (TSR-HDPR) features for multifunctional detection of complex physical and chemical stimuli.

## Introduction

Intelli-sense is becoming a leading frontier, finding exciting applications concerning health care^1–3^, antiterrorism^4^, food safety^5^, environmental monitoring^6^, Internet of Things^7^, etc. It is appealing to develop all-in-one multifunctional sensing systems that can guarantee the simultaneous sensing and differentiating of many physical and chemical stimuli^8,9^. Since common stimuli from environments are usually presented in an integrated format^10^, the main concern lies in the effective identification of each stimulus when using only one-sensing system. For this issue, array-based e-nose/e-tongue sensing approaches, analogous to mammalian sensation^11^, would work through discriminating a distinct pattern of responses generated by stimuli, leading to a fingerprint for their recognition. This approach depends on the interactions between multiple reporter elements and the stimuli of interest^12,13^. Typically, a traditional e-nose/e-tongue sensor array has a planar layout of substrate, in which different sensors made from various materials^14^ or chemically modified host material^15^ are mainly exposed to stimuli within a planar space at the same time. Although prominent progress has been made, considerable costs and sophisticated design with adequate chemical diversity (e.g., multiple reporter elements) to differentiate stimuli are often required in the fabrication of most planar sensor arrays. Additionally, some e-nose/e-tongue sensor arrays cannot always successfully differentiate complex physical and chemical stimuli due to some hard-to-distinguish crosstalk interferences among them, thus posing certain barriers to their wider applications. For these reasons, it is highly inspiring to develop novel sensor arrays with only one reporter element that nevertheless are multifunctional for the simultaneous sensing and distinguishing of complex physical and chemical stimuli, including temperature, relative humidity (RH), volatile organic compounds (VOCs), etc.

One way to overcome the challenges of e-nose/e-tongue sensor arrays for pattern discrimination between chemical stimuli is by linking them to time–space-resolved (TSR) separation techniques, such as gas chromatography (GC), which are used in differentiating subtle partitioning between a mobile phase (stimuli or analytes) and sequential stationary phases (substrates) during the diffusion/transfer process of analytes. This approach, however, is unsuitable for distinguishing physical stimuli (e.g., temperature). Furthermore, the timescale of the separation of compounds before entering the sensing area is slow. Notably, GC often needs large-size, sophisticated, expensive equipment; trained professionals; and high costs for maintenance, which inevitably limit its utilization for on-site analysis and large-scale application in resource-poor regions. To address this, it is highly desirable to develop portable, disposable, and cost-effective e-nose/e-tongue with TSR high-discriminative pattern recognition (TSR-HDPR) features for multifunctional sensing.

Herein, we present origami-based hierarchical electronics with TSR-HDPR features for multifunctional detection of complex physical and chemical stimuli. Using this concept enables the integration of a group of sensors based on conductive ink onto a substrate with hierarchical configuration and then responding to stimuli in a TSR mode. The resulting ink-loaded origami exhibits powerful TSR-HDPR sensory electronics for distinguishing multiple physical and chemical stimuli, even recognizing structural isomers and chiral enantiomers of VOCs.

## Results

### Formulation of bioinspired conductive P/G ink

The fabrication of the TSR origami sensors array starts with the formulation of bioinspired conductive ink. Conductive ink is one of candidates for this type of electronics due to its low-cost and compatibility with mass production^16^. Furthermore, it is more interesting to employ a functional ink that disperses well in environmental-friendly organic solvents, possess good adaptation and tailoring abilities, and readily interacts with substrates in a binder-free manner^17^. For our purposes, we developed a bioinspired conductive ink from a simple one-pot hydrothermal reduction of graphene oxide (GO) in dopamine (DA) as co-reductant (Supplementary Fig. 1). DA is a unique catechol-derived molecule mimicking mussel-adhesive proteins^18^. It can be oxidized and self-polymerized to form melanin-analogous polydopamine (PDA) for coating easily on many substrates with excellent affinity^18,19^. Thus DA works both as a reducing agent for GO and a capping agent to allow the as-synthesized reduced GO (rGO) for further surface modification^20^. Ethanol was chosen to transfer the resulting PDA@rGO into a homogenous P/G ink, due to ethanol’s low surface tension for substrate wetting and low boiling point for fast ink drying^17^. As a control, rGO and PDA were prepared under the same condition as P/G ink.

P/G ink is fully compatible with common substrates, including Kapton (commercially polyimide film), aluminum foil, inkjet paper, nitrile rubber, polydimethylsiloxane, and glass (Supplementary Fig. 2). Transmission electron microscopy (TEM), Raman spectroscopy, and scanning electron microscopy (SEM) were used to characterize GO, rGO, PDA, and P/G ink (Supplementary Fig. 3, 4, and 5). From the results, we can see that there are some porous structures in the films of rGO and PDA@rGO (Supplementary Figs. 3 and 5), and PDA@rGO has a smoother surface than rGO due to the coating of PDA. DA can influence the hydrothermal reduction of GO in terms of products’ morphology (Supplementary Fig. 5), and the deposited droplets of the above products showed a variety of spots (Supplementary Fig. 6). The deposited droplet of rGO produced obvious coffee-ring effect on the surface of untreated glass, whereas P/G ink recruits the synergistic benefits from PDA and GO for completely wetting the untreated glass surface via recirculating Marangoni flow to relieve the coffee ring effect^21^. Besides, P/G ink is conductive, like rGO (Supplementary Fig. 6b and d), and its sheet resistance is ~5.479 × 10^−4^ Ω•m.

**Fig. 1:**
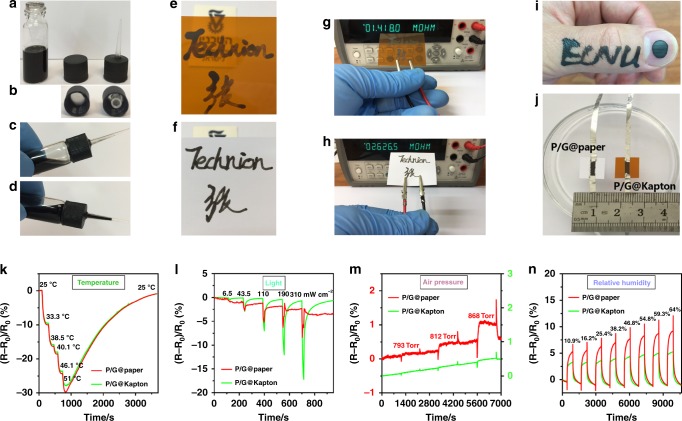
Formulation of bioinspired conductive ink for writing electronics and sensors. **a** A vial-based do-it-yourself (DIY) pen loaded with P/G ink. **b** The DIY pen tip is a 10-μL pipette microtip punched in a vial cap. **c**, **d** P/G ink can controllably flow in the DIY pen tip. **e**–**h** Conductive texts written on transparent Kapton film and paper. **i** P/G ink written on skin and fingernail as a conductive tattoo. **j** Image showing the sensors of the P/G@paper and P/G@Kapton. The initial resistance (*R*_0_) of the P/G@paper is 45.3 kΩ and of the P/G@kapton is 77.3 kΩ. **k**–**n** Resistance responses of the P/G@paper and P/G@Kapton to temperature (25–51 °C), light (6.5–310 mW cm^−2^), air pressure (793–868 Torr), and relative humidity (10.6–64%). Note: decreases in resistances of the P/G@paper and P/G@Kapton with the increasing temperatures or light powers can be attributed to the thermoconductive or photoconductive features of the P/G ink, whereas air pressure or relative humidity increase the resistances of the P/G@paper and P/G@Kapton

We confirmed the feasibility of P/G ink for writing flexible substrates (Fig. 1e, f). The written texts on Kapton and paper are conductive (Fig. 1g, f). Impressively, P/G ink can be written on skin or fingernail as a conductive tattoo (Fig. 1i), which is waterproof (Supplementary Movie 1) and wearable (>3 days). The waterproof capability of the P/G ink tattoo can be attributed to the strong affinity of mussel-inspired PDA matrix with the rGO^18,19^. Removal of the P/G ink tattoo can be achieved by ethanol. Concerns regarding the safety of the P/G ink were examined via cytotoxicity and blood tests. 293-cell cultured samples were exposed to 10–100 μg/mL of PDA@rGO for 24 h. Examination with WST-8 assay (Supplementary Fig. 7a) showed 102.7 ± 3.1% cell survival even upon exposure to 100 μg/mL of PDA@rGO. The time-course level of immunoglobulin E (IgE, one of the immune system markers) in the blood of young Sprague-Dawley rats showed no changes (or changes within the experimental error) when part of their (shaved) skin was covered with P/G ink (Supplementary Fig. 7b). These results indicate that the P/G ink is biocompatible and safe for use on skin.

Paper and Kapton were written with P/G ink and, respectively, linked with wires to form flexible resistors, namely, P/G@paper and P/G@Kapton (Fig. 1j). Both P/G@paper and P/G@Kapton showed quantitative and reversible resistance responses under temperatures ranging from 25 to 51 °C (Fig. 1k). They also had almost the same linear calibration curves (Supplementary Fig. 8a), giving a similar response regarding sensitivity, estimated to be ~1.1% per °C. These results indicate that the different substrates have negligible effect on the thermoconductivity of P/G ink. However, P/G@paper and P/G@Kapton responded differently to light exposure, air pressure, and RH (Fig. 1l–n). The P/G@paper responded more strongly to air pressure (Supplementary Fig. 8c) and RH (Supplementary Fig. 8d) than the P/G@Kapton in the same range, the responses of P/G@Kapton to air pressure were negligible. The response/recovery time of P/G@paper to air pressure was ~2 min (Fig. 1m), giving an estimated sensitivity of ~0.006% per Torr (Supplementary Fig. 8c). The response and recovery time of both P/G@paper and P/G@Kapton toward RH were ~9 and ~13 min, respectively, (Fig. 1n). The response sensitivity of P/G@paper and P/G@Kapton to RH was ~0.09% per RH and ~0.057% per RH (Supplementary Fig. 8d). A possible reason for these differences is that P/G@paper presents  a fibrous loose surface, because of the paper-based substrate (Supplementary Fig. 9), thus improving the responses toward pressure and RH^22^; however, P/G@Kapon has a compact surface due to a smooth Kapton substrate, which could provide more effective photoconductivity on exposure to light^23^ (Supplementary Fig. 8b). The response sensitivity of P/G@Kapon to light was ~0.051% per mW cm^−2^, which is greater than that of P/G@paper of ~0.019% per mW cm^−2^ (Supplementary Fig. 8b). Therefore, the sensory responses of functional P/G ink can be partly tuned toward diverse stimuli by using different substrates.

### P/G ink-loaded origami hierarchical sensor array (OHSA)

**Fig. 2:**
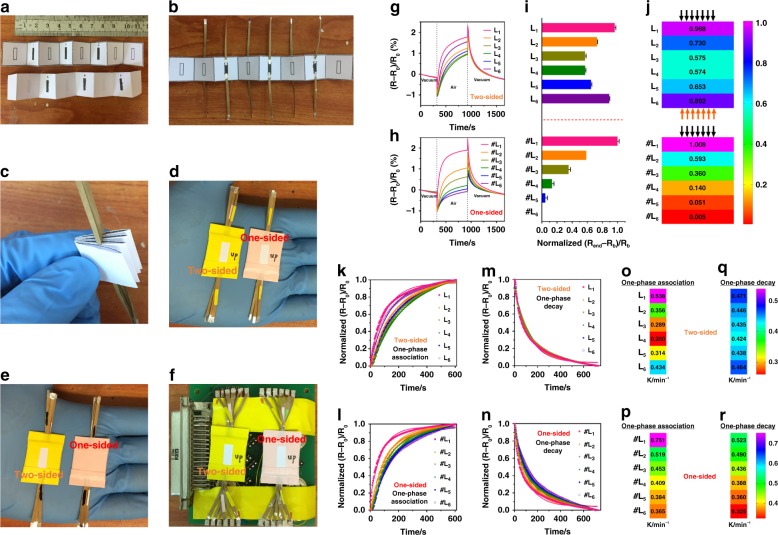
Devising and characterization of P/G ink-based origami hierarchical sensors array (OHSA). **a**–**f** Images describing the design of six-channel OHSA by one P/G ink integrated on a folding paper. **g**, **h** Time-course monitoring of the resistance responses in two-sided OHSA and one-sided OHSA upon alternately exposed to a vacuum and air. **i**, **j** Bars and color maps represent the layer-dependent (*R*_end_ − *R*_b_)/*R*_b_responses of two-sided OHSA and one-sided OHSA to air exposure in the two models, respectively. Data are presented as the mean ± SD. Kinetic analysis of the resistance responses of two-sided OHSA (**k**, **m**) and one-sided OHSA (**l**, **n**) to air exposure and vacuum clean, respectively. Color maps represent the layer-dependent rate constants (*K*) of two-sided OHSA (**o**, **q**) and one-sided OHSA (**p**, **r**) to air exposure and vacuum clean, respectively

P/G ink was written onto the fixed area of origami’s alternative six layers as the internal response indicators (Fig. 2a). The resultant lanes were linked with wires (Fig. 2b). The zigzag layers were folded (Fig. 2c) and sealed with tapes to form a one-sided or a two-sided OHSA (Fig. 2d, e). In this design, two-sided OHSA has two unsealed entrances through which direct exposure to stimuli can occur, allowing their permeation/diffusion into OHSA. On the other hand, one-sided OHSA has only one entrance. Owing to the highly ordered nature of the paper substrate, we assumed that this specific hierarchical layered configuration of OHSA would be an effective blocking framework as gradient generator for permeable/diffusible stimuli. Additionally, only one P/G ink used in OHSA would potentially yield distinguishable sensory responses toward permeable/diffusible stimuli with TSR-HDPR performances.

The resultant OHSAs were fixed on the electronic board for testing (Fig. 2f) in a program-controlled stainless-steel chamber by alternately being exposed to vacuum and air (Fig. 2g, h). The relative resistance change (i.e., (*R*_end_ − *R*_b_)/*R*_b_, where *R*_b_ stands for the base resistance at the beginning of air exposure and *R*_end_ is the resistance at the end of air exposure) was used to characterize these layer-dependent characteristics. On exposure to purified air from a commercial gas generator, the resistances of outer layers of OHSA increased faster than those of its inner layers, which could be indicative of the process of air permeation/diffusion (Fig. 2g, h). Interestingly, two-sided and one-sided OHSA were significantly different in the layer-dependent characteristics upon exposure to air (Fig. 2i). Since air in the chamber can bilaterally enter two-sided OHSA from its two entrances (Fig. 2d–f), there were almost symmetrical relative resistance changes among its six layers, which decreased from outer layers to inner layers in two directions (Fig. 2i, j). Although two-sided OHSA has two entrances, the relative resistance changes in L_1_ and L_2_ layers near the upper entrance were slightly higher from that of L_6_ and L_5_ layers beside the bottom entrance, which could result from the spatial differences and air flow directions (Fig. 2f). Owing to only one entrance for air permeation/diffusion, one-sided OHSA exhibited a sequential decrease of relative resistance changes from outermost layer to inner layers (Fig. 2i, j). Afterwards, there were also distinct time-dependent decreases in resistance among the different layers of these two OHSAs under a following vacuum clean of the air species on OHSAs’ layers (Fig. 2g, h). Consequently, the air/vacuum-triggered reversible layer-relevant resistance changes of OHSA can be attributed to the permeable/diffusible species (e.g., moisture, like Fig. 1n) in the air that interacted with the P/G ink loaded on hierarchical paper substrates. OHSA with several entrances can effectively tune the path of air permeation/diffusion with individual resistance response patterns (analogous to the TSR features of GC). Since the air-induced relative resistivity of OHSA saturated with time (Fig. 2g, h), we then evaluated the layer-relevant resistance changes of one-sided OHSA challenged with different air exposure times (Supplementary Fig. 10); the results revealed that different exposure time has little effect on the OHSA’s features. Considering that the features of the OHSA system may be due to the way it is packed (e.g., the space between layers might be different), we further fabricated 21 one-sided OHSA in the same way and assessed the reproducibility of their features. The results showed that there is robust reproducibility regarding their layer-involved resistance changes (i.e., relative standard deviation values of (*R*_end_ − *R*_b_)/*R*_b_ are all <12%) (Supplementary Fig. 11). Apart from OHSA with symmetrical zigzag folding, we also fabricated OHSA with layer-by-layer strategy, in which an individual layer was cut at the fold and stacked on the top of one other. Owing to the analogous hierarchical structure, the response of layer-by-layer OHSA is like that of the zigzag folding OHSA (Supplementary Fig. 12). Therefore, the OHSA design can be potentially extended to other strategies. Compared to the layer-by-layer strategy, zigzag folding would be a more controllable and integrated framework for the encapsulation of OHSA for further applications.

The time-dependent resistance responses of OHSA layers were then used to determine hierarchical kinetic parameters by fitting with equations for binding kinetics to air exposure and vacuum clean. Vacuum clean means that the exposure chamber is under a pressure of 0.06 Torr, which removes the air species absorbed on the P/G ink films. The raw resistance data of the different layers of OHSA were normalized and the curves were divided into two parts: the processes of (1) air exposure (Fig. 2k, l), and (2) vacuum clean (Fig. 2m, n). A one-phase association was the best fit for the OHSA layers exposed with air and a one-phase decay for the succedent dissociation by vacuum clean, respectively (Fig. 2k–n). The coefficients of goodness of fit (i.e., *R*-square) were largely around or >0.98. The resulting OHSA’s response kinetic parameters regarding rate constant (*K*) were then obtained (Fig. 2o–r). The *K* values of the two-sided OHSA decreased symmetrically from the outer layers to the inner layers in two directions and that of one-sided OHSA decreased sequentially from the outermost layer to the inner layers. The change in trends of *K* values were well consistent with the abovementioned relative resistance changes, indicating that both the relative resistance changes and *K* values can provide quantitative descriptions of stimuli-responsive OHSA’s layer-relevant sensory behaviors.

Theoretical modeling was also performed to investigate the mass transport through OHSA. We treated the sensor array as a porous block of material that initially is in vacuum (within a chamber) and is suddenly exposed to a step-rise in pressure. Owing to the abrupt rise in pressure, it is reasonable to assume that the chamber and the porous material are immediately filled with the sample gas. For simplicity, we assumed that the gas contains a single impurity of which the small amount initially entering the porous material is mostly and immediately adsorbed. Thus at time = 0 the gas phase in the chamber contains a background concentration of the impurity and the gas phase within the pores contains very little impurity and is in equilibrium with a small amount adsorbed onto the porous medium; the system is at uniform pressure. In this case, we can treat the problem as a diffusion–adsorption problem described by the following non-dimensional partial differential equation (PDE) and initial and boundary conditions:

1 $${\hskip 30pt}\frac{{\partial c}}{{\partial t}} = \frac{{\partial ^2c}}{{\partial x^2}} {\hskip 20pt} t > 0,{\hskip 18pt}0 \le x \le 1\hfill$$

2 $${\hskip 30pt}{c = 0{\hskip 37pt}t = 0,{\hskip 11pt} 0 \le x \le 1}{\hskip 0pt}\hfill$$

3 $$ {\hskip 30pt}{c = H\left( t \right){\hskip 23pt} t > 0, {\hskip 18pt} x = 0}\hfill$$

4 $${\hskip 30pt}\frac{{\partial c}}{{\partial x}} = 0{\hskip 29.5pt} t > 0,{\hskip 18.5pt} x = 1\hfill$$

Here the dimensionless concentration of impurity is defined as $c \equiv \frac{{\left( {\tilde c - \tilde c^ \ast } \right)}}{{\tilde c_0}}$, where $\tilde c$ is the dimensional impurity concentration in the gas phase, $\tilde c^ \ast$ is the small level of dimensional impurity concentration initially in the pores after the initial quick adsorption (immediately following the sharp rise in pressure), and $\tilde c_0$ is the background impurity concentration in the chamber (assumed to be constant, i.e., in the model, the chamber is assumed to be infinitely large compared to the porous medium). The dimensionless time is normalized using a characteristic time given by: $t_c = \left[ {1 + \frac{{\left( {1 - \varepsilon } \right)}}{\varepsilon }a} \right]\frac{{L^2}}{{\left( {D\varepsilon /\tau ^2} \right)}}$, where *ε* is the porosity, *L* is the thickness of the porous block of material in the one-sided case and half the thickness in the two-sided case, *D* is the gas phase diffusion coefficient of the impurity, *τ* is the tortuosity of the porous medium and *a* (assumed constant for simplicity) is the derivative of the adsorption isotherm (concentration of adsorbed material) with respect to the gas phase concentration, i.e., *a* = *dq*/*d*$\tilde c$, where *q* is the dimensional concentration of adsorbed material (assumed at equilibrium with the gas phase in the pores). Finally, the dimensionless distance *x* is normalized with *L* and *H*(*t*) as the Heaviside step function. The above PDE, initial condition, and boundary conditions have an analytical solution^24^:

5 $$c\left( {t,x} \right) = \frac{4}{\pi }\mathop {\sum }\limits_{n = 0}^\infty \frac{{\left( { - 1} \right)^n}}{{\left( {2n + 1} \right)}}\cos \left[ {\left( {\frac{{2n + 1}}{2}} \right)\pi \left( {1 - x} \right)} \right]\\ \times\left\{ {1 - {\mathrm{Exp}}\left[ { - \left( {\frac{{2n + 1}}{2}} \right)^2\pi ^2t} \right]} \right\}$$

Plots of this function for the six (dimensionless) sensor positions within OHSA (porous block of material) are provided for the one-sided and two-sided cases. Notice that, in the two-sided case, due to symmetry, only half the system is considered and hence the last two sensors are, in a sense, at the same positions as sensors two and three (Supplementary Fig. 13). Finally, these plots in Supplementary Fig. 13 are qualitatively similar to the above experimental observations in Fig. 2g, h.

### Multifunctional monitoring of multiple stimuli using OHSA

**Fig. 3:**
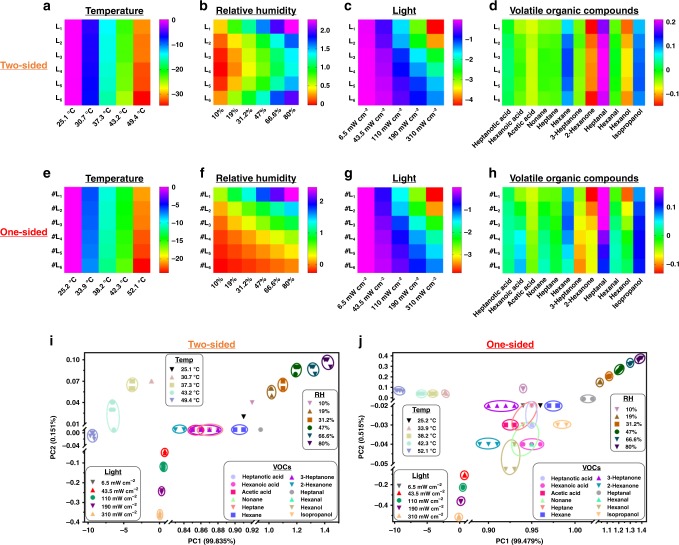
Origami hierarchical sensor array (OHSA) for the discrimination of multiple chemical and physical stimuli. Color maps represent the responses of two-sided and one-sided OHSA to **a**, **e** temperature (~25–50 °C), **b**, **f** relative humidity (10–80%), **c**, **g** light (6.5–310 mW cm^−2^), and **d**, **h** VOCs (10 ppm), respectively. Two-dimensional canonical score plots of two-sided **i** and one-sided **j** OHSA’s responses to the multiple stimuli as indicated

Two-sided OHSA and one-sided OHSA were, respectively, challenged with different stimuli, including air pressure, temperature, RH, light, and VOCs (Fig. 3a–h, Supplementary Fig. 14-18 and Supplementary Table 1). Owing to the blocking effect of outer capsulation, P/G@paper layers inside these two OHSAs were relatively stable when exposed to air pressure at from 793 to 868 Torr (Supplementary Fig. 19) compared to bare P/G@paper (Fig. 1m, Supplementary Fig. 8c). There was no obvious difference in our test settings between two-sided OHSA and one-sided OHSA in response to temperature (Fig. 3a, e) and light exposure (Fig. 3c, g) because of their unidirectional transmission; that is, the resistance responses of these two OHSA layers had monodirectional gradient tendencies toward temperature (Supplementary Fig. 14) and light exposure (Supplementary Fig. 16). Regarding the monitoring of RH and VOCs, two-sided OHSA presented nearly symmetrical resistance responses toward these stimuli from the outer layers to the inner layers (Fig. 3b, d, Supplementary Fig. 15a-b and 17a); by contrast, one-sided OHSA showed one-way responses (Fig. 3f, h, Supplementary Fig. 15e-f and 17c). These diverse results can be attributed to the random permeation/diffusion of RH and VOCs into the two entrances of two-sided OHSA and one entrance of one-sided OHSA (Fig. 2d–f). The layer-dependent response sensitivity of the two OHSAs to temperature, RH, light, and VOC (Heptanal as a model) are summarized in Supplementary Table 2, and their analytical performances were compared to some reported sensors. For example, owing to the excellent thermal conductivity of rGO as a component, our OHSAs have more sensitivity to temperature (~1.272% per °C of two-sided OHSA) than that of 1-decanethiol-capped Au–monolayer-capped metal nanoparticle sensor (0.65% per °C)^10^. In addition, they have comparable sensitivity in the monitoring of Heptanal (~0.02% per ppm of two-sided OHSA), in contrast to that of maleic acid-doped polyaniline sensor (~0.03% per ppm)^9^.

Principal component analysis (PCA)^25^ was used to differentiate the resistance response patterns of OHSA against each stimulus. The results show that the two OHSAs can discriminate each stimulus (temperature, RH, light, or VOCs) with different levels (Supplementary Fig. 14c, 14g, 15c, 15g, 16c, 16g, 17b, 17d, 18c, and 18g). The resultant PC1 can be used for quantitatively measuring different degrees of each stimulus (Supplementary Fig. 14d, 14h, 15d, 15h, 16d, 16h, 18d, and 18h). Moreover, the canonical resistance-response patterns from all the above stimuli were fully classified into the independent area of two-dimensional (2D) PCA plot (Fig. 3i, j). Notice that there is some overlap between the resistance-response patterns from the tested VOCs in the 2D PCA plots using both the OHSAs; therefore, we have employed three-dimensional (3D) PCA plots to better illustrate the  differences in the VOC-related resistance-response patterns (Supplementary Fig. 20). As a result, most of the tested VOCs (including acids, alkanes, ketones, aldehydes, and alcohols listed in Supplementary Table 1) were differentiated using two-sided OHSA (Supplementary Fig. 20a), and all the tested VOCs were clearly distinguished using one-sided OHSA (Supplementary Fig. 20b), suggesting that one-sided OHSA would has stronger discrimination toward VOCs due to its unique one entrance for the random permeation/diffusion of VOCs. Accordingly, OHSA can also be a smart e-nose/e-tongue for simultaneous sensing and differentiating between complex stimuli. For example, three structurally similar VOCs (i.e., 2-Hexanone, Hexanal, and Heptanal, 10 ppm) could be effectively separated into their individual locations. To validate the TSR-HDPR features of OHSA, we exposed the two OHSAs to these three VOCs mixtures at different mass ratios (2-Hexanone:Hexanal:Heptanal = 4:4:2, 4:2:4, 2:4:4; all in ppm) (Supplementary Fig. 21). Unique clusters were distinguishable among these mixtures in the 3D PCA plots using both two-sided OHSA (Supplementary Fig. 21b) and one-sided OHSA (Supplementary Fig. 21c).

Considering that many stimuli usually change simultaneously, discrimination between various physical and chemical stimuli in complex environments is difficult to achieve with a sensor array based solely in chemical interactions. A pre-calibrated array of chemical and physical sensors would allow overcoming this challenge. Sensors immune to chemical analytes and solely dedicated for measuring physical parameters (such as temperature, RH) could be used to assess the physical conditions. As a proof of concept, we examined the discrimination of VOCs (10 ppm Hexanol, 10 ppm Heptanal, and the mixture of 5 ppm Hexanol + 5 ppm Heptanal) at different temperatures (20 °C and 30 °C) using our OHSA, respectively (Supplementary Fig. 22). The results prove that the canonical resistance-response patterns from the aforesaid VOCs can, respectively, be easily differentiated using our one-sided OHSA at both temperatures. Since the second principle component (PC2) and the third principle component (PC3) of the 3D PCA plots were <40%^13^ (Supplementary Fig. 22a), it was reasonable and acceptable to adopt the first principle component (PC1) to depict the pattern of aforesaid VOC samples under different temperatures (Supplementary Fig. 22b), which shows that these VOC samples at different temperatures can be mapped for tracing each parameter alone, verifying the robustness of our OHSA for use in complex environment.

**Fig. 4:**
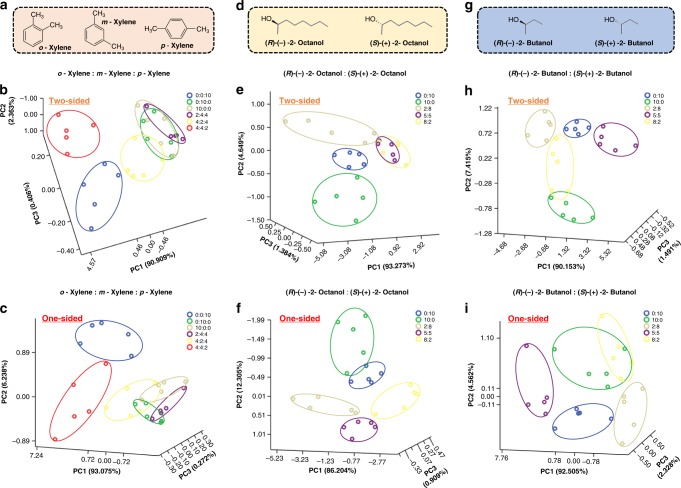
Identification of structural isomers and chiral recognition of enantiomers. **a** Structural formula of Xylene isomers. Three-dimensional (3D) canonical score plots for two-sided origami OHSA (**b**) and one-sided OHSA (**c**) against Xylene isomers (10 ppm) and their mixtures at the indicated different mass ratios, respectively. Structural formula of 2-Octanol enantiomers (**d**) and 2-Butanol enantiomers (**g**). 3D canonical score plots for two-sided OHSA (**e**, **h**) and one-sided OHSA (**f**, **j**) against 2-Octanol enantiomers (10 ppm) and 2-Butanol enantiomers (10 ppm) and their mixtures at the indicated different mass ratios, respectively

The successful pattern recognition of 10 ppm 2-Hexanone and Hexanal (two position isomers with the same molecular formula, illustrated in Supplementary Fig. 21a, and 20) using OHSA would present the possibility of identifying more structural isomers of VOCs. To demonstrate this point, three isomers of Xylene (*o*-Xylene, *m*-Xylene, and *p*-Xylene), important chemical intermediates in industries but harmful substances to human health^26^, were chosen as model analytes and monitored using OHSA (Fig. 4a). One set of Xylene isomer samples was created by spiking *o*-Xylene, *m*-Xylene, and *p*-Xylene at various mass ratios (*o*-Xylene:*m*-Xylene:*p*-Xylene = 10:0:0, 0:10:0, 0:0:10, 4:4:2, 4:2:4, 2:4:4; all in ppm). From the 3D PCA plots of two-sided OHSA (Fig. 4b) and one-sided OHSA (Fig. 4c), 10 ppm Xylene isomers and their mixtures at different mass ratios can be characteristically distinguished and detected at different locations according with their mass ratios. We have used density functional theory (DFT) molecular modeling of P/G after exposure to VOCs to explain the mechanism of resistance changes of the sensor and how to use them for discrimination between isomers (*m*-Xylene, *o*-Xylene, and *p*-Xylene). According to the formulation of P/G ink, PDA is believed to self-polymerize from DA during hydrothermal reduction of GO. The non-covalent interaction between PDA and rGO seems to affect the HOMO-LUMO level of P/G ink during exposure. We have used the Gaussian software to examine these complexes at APFD/6-31G* level. The result in Supplementary Table 3 shows that the highest occupied molecular orbital (HOMO)–lowest unoccupied molecular orbital (LUMO) levels (and band gap to some extent) vary differently for different isomers. This could explain the resistance change of the sensor in the experiment with different Xylene isomers, as HOMO–LUMO levels of complexes varied with respect to the Fermi level of the metal electrode of the sensor. Seeing that PC2 and PC3 of the 3D PCA plots were <40% (Fig. 4b, c), PC1 can be also used to profile the pattern of Xylene isomer samples. As shown in Supplementary Fig. 23, the two OHSAs were completely sensitive in mapping these isomers in predetermined mixtures for tracing each isomer alone, which is comparable to the performance of GC.

There is another main form of isomers, namely, enantiomers (also known as stereoisomers with chirality). A simple, sensitive technique for discriminating between enantiomers of a chiral molecule is paramount in many fields of chemistry, biotechnology, and medicine^27^. Two groups of enantiomers of 2-Octanol and 2-Butanol (Fig. 4d, g) were selected to check the feasibility of OHSA for chiral recognition of VOCs’ enantiomers. One set of 2-Octanol samples was prepared by mixing (*R*)-(+)-2-Octanol and (*S*)-(–)-2-Octanol at various mass ratios ((*R*)-(+)-2-Octanol: (*S*)-(–)-2-Octanol = 10:0, 2:8, 5:5, 8:2, 0:10, all in ppm), and the other set of 2-Butanol samples was made by spiking (*R*)-(+)-2-Butanol and (*S*)-(–)-2-Butanol at different mass ratios as indicated above. The data shows conclusively that there were distinguishable and separate groups in the 3D PCA plots of OHSA corresponding to the tested enantiomers and their mixtures at their different mass ratios (Fig. 4e, f, h, i). The resultant PC1 from the 3D PCA plots of OHSA also describes the pattern of the tested enantiomer samples and effectively locate/match these enantiomers at various mixtures for determining each enantiomer in them (Supplementary Fig. 24). These interesting findings show that our OHSA is also competent for chiral recognition of VOCs’ enantiomers, which is probably the first time such data have been reported about the chiral recognition of VOC enantiomers.

To illustrate the mechanism of OHSA for distinguishing VOC isomers and enantiomers, one P/G ink-coated quartz crystal microbalance (QCM) sensor was equipped (Supplementary Fig. 25a and b) and three P/G@paper sensors were configured with different layers of paper from 0 to 3 as outside covers (Supplementary Fig. 26a). The P/G ink-coated QCM sensor was exposed to Xylene isomers and 2-Octanol enantiomers (Supplementary Fig. 25c). The results demonstrate that the absorption or diffusion of such VOC molecules into the film of P/G ink results in mass changes, but there is negligible mass discrimination between VOC isomers and enantiomers. Three P/G@paper sensors covered with different layers of paper were also challenged with Xylene isomers (Supplementary Fig. 26b-d) and 2-Octanol enantiomers (Supplementary Fig. 26f-h). The P/G@paper sensor without an outside cover showed little different (*R*_end_ − *R*_b_)/*R*_*b*_ responses between the Xylene isomers (Supplementary Fig. 26b and e), whereas the P/G@paper sensors covered with 1 layer or 3 layers of paper can have more distinguishable and layer-dependent (*R*_end_ − *R*_b_)/*R*_b_ responses between Xylene isomers (Supplementary Fig. 26c-e). Additionally, the P/G@paper sensors covered with outside layers can show chiral discrimination toward 2-Octanol enantiomers as the number of layers increases (Supplementary Fig. 26f-i). These results prove that our OHSA design represents a key and effective mass transport for discriminating structural isomers and enantiomers through its unique TSR-HDPR features.

**Fig. 5:**
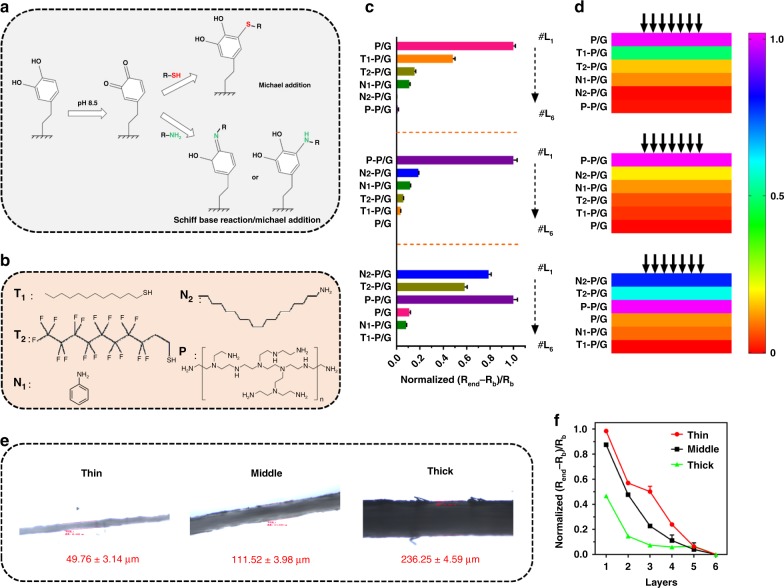
Tuning the performances of origami hierarchical sensor array (OHSA). **a** Schematic illustration of the typical chemical reactions of polydopamine with thiol-containing compounds (via Michael addition) and amino-containing compounds (Schiff base reaction/Michael addition). **b** Structural formula of the chemical ligands used for the functionalization of P/G ink. Bars (**c**) and color maps (**d**) represent the (*R*_end_ − *R*_b_)/*R*_b_ responses of one-sided OHSA written with different functional P/G inks in different orders toward air exposure, respectively. Data are presented as the mean ± SD. **e** Microscopic measurement of papers of different thickness (thin, middle and thick). **f** Plots represent the (*R*_end_ − *R*_b_)/*R*_b_ responses of one-sided OHSA by using these papers in diverse tracks toward air exposure. Data are presented as the mean ± SD

Our proposed OHSA-based smart hierarchical design could be further extended for possessing more characteristics by adjusting the configuration of origami, e.g., by writing with other functional inks. Our P/G ink is suitable for the further development of many of functional inks because the catechol in its PDA matrix is reactive and can be readily oxidized into quinone under basic conditions. This can react with thiol-containing chemicals via a Michael addition pathway and amine-containing chemicals through a Schiff base reaction/Michael addition in a facile mix-and-react manner at room temperature^19^, as depicted in Fig. 5a and Methods. Five chemicals including two thiol-containing small molecules (T_1_, T_2_), two amine-containing small molecules (N_1_, N_2_), and one amine-containing polymer (P) (Fig. 5b) were chosen as ligands for engineering the surface of P/G ink. SEM was used to characterize the surface morphology of the dried inks (Supplementary Fig. 27a-f). The results indicate that the different ligands can significantly tune the surface structure of dried inks, and the resultant surfaces have distinct water contact angle (Supplementary Fig. 27g-l). The results confirm that our P/G ink is readily and universally modified with ligands through its PDA’s chemical reactivity to create various functional P/G inks with tailored chemical diversity. Then, as proof of concept, three one-sided OHSAs have been configured by writing with these functional inks in different orders (Fig. 5c). Figure 5c, d show that different functional inks located in different layers can tune the layer-dependent characteristics of relative resistance changes to air exposure, which is unlike that of P/G ink-loaded one-sided OHSA (Fig. 2i, j). Note that the selected chemical ligands play an important role for providing OHSA with the resultant diverse characteristics. In addition, paper porosity affects how thoroughly and quickly inks are absorbed into a paper primarily by capillary action and the length of time it takes for air to permeate/diffuse a paper or the rate of the passage of air through it. Thus another potential way to tune the configuration of origami for regulating its TSR-HDPR characteristics can be adopted by using porous papers with different qualities. In response to this, another one-sided OHSAs have been fabricated by using three types of paper (thin, middle, and thick, with different porosity, Fig. 5e) as substrates written with the same ink. Figure 5f shows that the thickness of the paper as substrates can also modulate the layer-related characteristics of relative resistance changes to air exposure, thinner paper giving rise to more extensive responses. We also examined the effect of the amount of ink deposited on the substrate on the resistivity readings of OHSA (Supplementary Fig. 28), confirming that different ink levels can change the thickness of the film for also regulating the layer-related characteristics of relative resistance changes to air exposure. From the above, the integration of OHSA-based hierarchical design and chemical engineered inks, diversified paper substrates, or various amounts of ink applied would result in more TSR-HDPR electronics for widespread applications.

## Discussion

We have introduced the first practical invention of fundamental ingenious OHSA, written with a conductive ink for multifunctional sensory application, based on its newly TSR-HDPR features. OHSA is low cost, yet powerful, in responding to multiple chemical and physical stimuli and has competence in the simultaneous sensing and differentiation of complex stimuli, especially in discriminating VOCs’ isomers and chiral recognition of VOCs’ enantiomers.

## Methods

### Preparation of GO

Briefly, 2 g graphite was put into a mixture of concentrated H_2_SO_4_ (80 mL), K_2_S_2_O_8_ (7 g), and P_2_O_5_ (5 g). The solution was heated to 80 °C for 5 h. The mixture was diluted with water and left overnight, before the product was collected by centrifugation. This solid was re-oxidized by another oxidation stage. For this purpose, the as-prepared product, as well as NaNO_3_ (1 g), was mixed with H_2_SO_4_ (95%, 48 mL) in a 250-mL flask. The mixture was stirred for 30 min in an ice-bath. Six grams of KMnO_4_ was gradually added to the suspension under vigorous stirring. The ice-bath was removed and the mixture stirred at 35 °C for 5 h. Deionized water (60 mL) was slowly added to the paste with vigorous stirring. The reaction temperature was increased to 98 °C, and the sample was maintained for 2 h at this temperature. Finally, H_2_O_2_ (30%, 20 mL) was added to the mixture. The mixture was washed with HCl (5% w/v) and deionized water several times to obtain GO, which was duly collected and dried under vacuum at 60 °C for 6 h.

### Preparation of PDA-modified graphene ink (P/G ink)

In a typical synthesis, 25 mg dopamine hydrochloride dissolved in 20 mL of water was mixed with 5 mL of 5 mg/mL GO (pH adjusted to 8.5). The mixture was kept in a Teflon-lined autoclave at 160 °C for 12 h. After hydrothermal reaction, the autoclave was cooled to room temperature and the product was washed in deionized water and ethanol several times, collected by centrifuge, and re-dispersed in ethanol for further use. It is named as P/G ink. As a control, rGO (only GO as reactant) and PDA (only DA as reactant) were prepared by the same method.

### Fabrication of OHSA

As depicted in Supplementary Fig. 29, a template was designed on PowerPoint with 11 detached columns, and each column has 8 squares with a size of 2 × 2 cm. There is a 0.5 × 1 cm size strip in the middle of every square area for writing ink. The template was then printed on a paper using printer. The printed paper template was cut into 11 columns, and every column could be alternatively folded into one symmetrical zigzag origami according to the lines between eight squares. P/G ink was written alternatively on the middle strip areas. After the ink had dried, the ink lanes were linked to aluminium foil strips (partly wrapped with tapes to avoid interconnections) using conductive silver paste at the contact. The resulting zigzag origami was folded into a square shape and sealed with tapes to form the OHSA. For two-sided OHSA, there are two entrances on each side without tape sealing, and for one-sided OHSA, there is one entrance on only one side (Fig. 2d–f).

### Preparation of ligand-modified P/G inks

P/G ink was dispersed in ethanol and the pH was adjusted to ~8.5 using alkaline ethanol solution. Thiol or NH_2_-containing compounds (T_1_:1-Dodecanethiol, T_2_:1 H,1 H,2 H,2H-Perfluorodecanethiol, N_1_:Aniline, N_2_:Oleylamine, and P:Polyethyleneimine, shown in Fig. 5b) were, respectively, mixed with P/G ink and the mixtures were kept for 12 h with gentle stirring. They were washed with ethanol several times, collected by centrifuge, and re-dispersed in ethanol for further use. They were named T_1_-P/G ink, T_2_-P/G ink, N_1_-P/G ink, N_2_-P/G ink, and P-P/G ink, respectively.

### Computational methods

Gaussian 09 software was used to calculate the molecular structure, the HOMO, and LUMO energy of complex formation of rGO-PDA with each Xylene isomer, using a DFT method with dispersion correction at the APFD/6-31G* level.

## Supplementary information

Supplementary Information

Peer Review

Description of Additional Supplementary Files

Supplementary Movie 1

Source Data

## Data Availability

The data that support the findings of this study are available from the corresponding author upon reasonable request.
