# Supplementary material for: Time-space-resolved origami hierarchical electronics for ultrasensitive detection of physical and chemical stimuli
Source: Nat Commun. 2019 Mar 8;10:1120. doi: 10.1038/s41467-019-09070-8 (PMC6408588; doi:10.1038/s41467-019-09070-8)
Supplement: Supplementary file 1 — Supplementary Information [file 41467_2019_9070_MOESM1_ESM.pdf]

Supplementary Information:

Time-Space-Resolved Origami Hierarchical Electronics for Ultrasensitive Detection of Physical and Chemical Stimuli

Min Zhang[1,2], Jiaxing Jeccy Sun[1], Muhammad Khatib[1], Zi-Yang Lin[2], Zi-Han Chen[2], Walaa Saliba[1], A'laa Gharra[1], Yehu David Horev[1], Viki Kloper[1], Yana Milyutin[1], Tan-Phat Huynh[3], Simon Brandon[1], Guoyue Shi[2], and Hossam Haick[1]*

[1] *Department of Chemical Engineering and Russell Berrie Nanotechnology Institute, Technion - Israel Institute of Technology, Haifa 320003, Israel.*

[2] *School of Chemistry and Molecular Engineering, Shanghai Key Laboratory for Urban Ecological Processes and Eco-Restoration, East China Normal University, 500 Dongchuan Road, Shanghai 200241, China.*

3 Laboratory of Physical Chemistry, Faculty of Science and Engineering, Åbo Akademi University, Porthaninkatu 3-5, FI-20500 Turku, Finland.

[*] *Correspondence and requests should be addressed to H.H. (E-mail: hhossam@technion.ac.il)*

Supplementary Methods

Chemicals. Synthetic graphite power was purchased from BDH chemicals Ltd. (Poole, England). Dopamine hydrochloride, 1-Dodecanthiol, 1H,1H,2H,2H-Perfluorodecanethiol, aniline, oleylamine, polyethyleneimine (branched, average Mw ~25,000 by LS) and other chemicals were ordered from Sigma-Aldrich, Inc. All chemicals used in the presented work were obtained from commercial sources and directly used without further purification. The substrates, including Kapton, aluminium foil, nitrile gloves, PDMS, glass and Teflon, were cleaned with ethanol prior to use, and no other surface treatment was used. Commercially available A_4 inkjet papers (80 g/m^2, thickness = 93.57 ± 3.45 μm, porosity = 41.5%) and other papers with different qualities (Thin: 49.76 ± 3.14 μm, porosity = 75.3%; Middle: 111.52 ± 3.98 μm, porosity = 62.6%; Thick: 236.25 ± 4.59 μm, porosity = 58.5%) were used as origami substrates.

Instrumentation. Contact angle measurements were performed using the ramé-hart Model 200 Standard Contact Angle Goniometer. The photoconductivity was measured by a Keithley meter under illumination by a full-spectrum LED chip (https://www.ebay.com/itm/Full-Spectrum-High-Power-LED-Chip-Grow-Light-3W-100W-380-840NM-FUll-spectrum-/262764518753?var=&hash=item3d2dfc5d61). Raman spectra were monitored using a WITec CRM200 confocal Raman microscope. Thorlabs S310C system was used to calibrate the optical power density (mW cm^{-2}) of the full-spectrum high power LED chip. For temperature measurement, the origami-inspired hierarchical sensor array (OHSA) was fixed on a temperature-controlled hot plate and a thermometer was also used as the calibration. For the measurement of humidity, air pressure or VOCs, OHSA was linked to a designed circuit board and incubated in a stainless-steel chamber for exposing to stimuli controlled by a program-controlled gas generator system (MCZ, Umwelt Technik). Morphologies of samples were characterized by scanning electron microscopy (SEM, S4800, Hitachi) and transmission electron microscopy (TEM, JEM-2011F, JEOL). Mercury intrusion porosimetry tests were carried out using a Micromeritics Autopore IV 9500 apparatus (Micrometics, Norcross, GA), which operates at pressures between 0.10 to 60000.00 psia, and data acquisition and calculation of results were done using the AutoPore IV 9500 software, version 1.07. Time-course of resistance changes were recorded by using a Keithley 2701 Ethernet Multimeter/Data acquisition system. The sheet resistances of P/G ink were determined using a four-point probe nanovoltmeter (ST2258C, Suzhou lattice electronic co., Ltd, China), these measurements were repeated 3 times at 25 °C, and the results were averaged.

Quartz crystal microbalance (QCM) assay. QCM is a piezoelectric mass-sensing device that has the ability to measure very small mass changes, down to a fraction of a monolayer or a single layer of atoms. QCM evaluates a mass per unit area by measuring the change in frequency (δ_f) of a quartz crystal resonator that is disturbed by the addition or removal of a small mass (Δm) at the surface of the acoustic resonator. The relationship between Δm and δ_f is described by the Sauerbrey's equation: $\Delta m = - C_f A \delta_f$, where C_f is the mass sensitivity (= 1.104 ng cm^{-2} Hz^{-1}) and A is the active area of the QCM resonator (= 0.2 cm^2). In the current study, one P/G ink-loaded gold-plated QCM resonator (5 mm in diameter; 20 MHz resonant frequency) was used to explore changes in the mass of the P/G ink film caused by exposures to the VOC analytes.

Data collection and analysis. OHSA used in this work responded to the stimuli by changing the resistance. The relative resistance changes were used to evaluate sensors' performances to different stimuli. For temperature sensing, $(R\text{-}R_0)/R_0$ was calculated as the feature to different temperatures, where R_0 is the initial resistance before the testing, and R is the resistance challenged with varying temperature. For monitoring of other stimuli (light exposure, air pressure, relative humidity, and VOCs), $(R_{end}\text{-}R_b)/R_b$ was calculated as the feature to these different stimuli, where R_b is the base resistance at the beginning of a new stimuli and R_{end} is the resistance at the end of every stimuli. These features were used to create a statistical model to distinguish between different groups via principal component analysis (PCA). IBM SPSS 22.0 software was used to process PCA. To determine the kinetic parameters of time-dependent resistance responses in OHSA layers, the raw resistance data were normalized and the curves were divided into two parts (one is the process of air exposure, the other is vacuum treatment), which were respectively fitted with nonlinear regression (viz. one-phase association and one-phase decay indicated below) using GraphPad Prism 7.0 software (San Diego, CA). After that, rate constant (K) values at different layers were obtained. GraphPad Prism 7.0 software was used to perform all the data plotting.

- **One phase association equation** (1) was used to describe the pseudo-first order association kinetics of the interaction between an analyte (each permeable/diffusible stimulus) and its receptor (each OHSA layer). During each time interval a certain fraction of the unoccupied receptors become occupied. But as time advances, fewer receptors are unoccupied so fewer analyte bind and the curve levels off (*Ref*: https://www.graphpad.com/guides/prism/7/curve-fitting/index.htm?REG_Exponential_association.htm).

$$Y = Y_0 + (Plateau\text{-}Y_0)*(1\text{-}\exp(\text{-}K*X)) \quad (1)$$

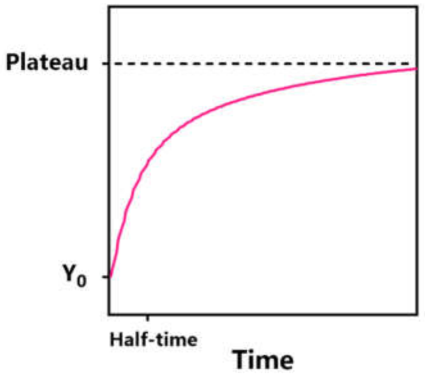

Y_0 is the ***Y*** value when ***X*** (time) is zero, expressed in the same units as ***Y***.

Plateau is the ***Y*** value at infinite times, expressed in the same units as ***Y***.

K is the rate constant, expressed in reciprocal of the ***X*** axis time units.

Tau is the time constant, expressed in the same units as the ***X*** axis. It is computed as the reciprocal of ***K***.

Half-time is in the time units of the ***X*** axis. It is computed as ln2/***K***.

■ **One-phase decay equation (2)** is an exponential decay equation modeling many chemical and biological processes. Typically, when analytes (each permeable/diffusible stimulus) dissociate from receptors (each OHSA layer), the number of molecules that dissociate in any short time interval is proportional to the number that were bound at the beginning of that interval. Equivalently, each individual molecule of analyte bound to a receptor has a certain probability of dissociating from the receptor in any small-time interval. That probability does not get higher as the analyte stays on the receptor longer (*Ref*: https://www.graphpad.com/guides/prism/7/curve-fitting/index.htm?REG_Exponential_decay_1phase.htm).

$$Y = (Y_0 - Plateau)*\exp(-K*X) + Plateau \quad (2)$$

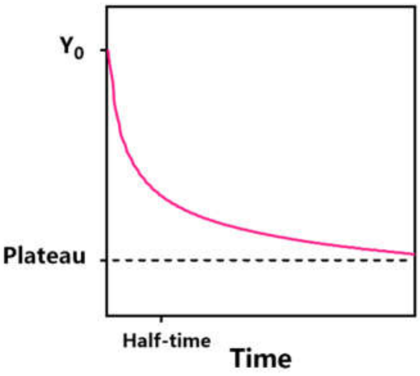

Y_0 is the *Y* value when *X* (time) is zero, expressed in the same units as *Y*.

Plateau is the *Y* value at infinite times, expressed in the same units as *Y*.

K is the rate constant, expressed in reciprocal of the *X* axis time units.

Tau is the time constant, expressed in the same units as the *X* axis. It is computed as the reciprocal of *K*.

Half-time is in the time units of the *X* axis. It is computed as ln2/*K*.

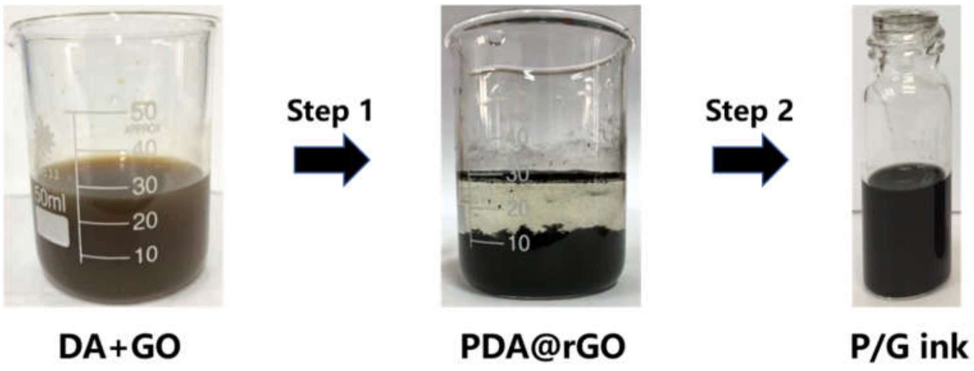

Supplementary Fig. 1 Flow diagram shows the fabrication process of the bioinspired P/G ink. **Step 1**: a brown yellow solution of DA+GO was exposed to a hydrothermal reaction in a Teflon-lined autoclave at 160 °C for 12 h, and then black PDA@rGO sediment formed. **Step 2**: the resultant product was washed by deionized water and ethanol several times, collected by centrifuge, and re-dispersed in ethanol as P/G ink.

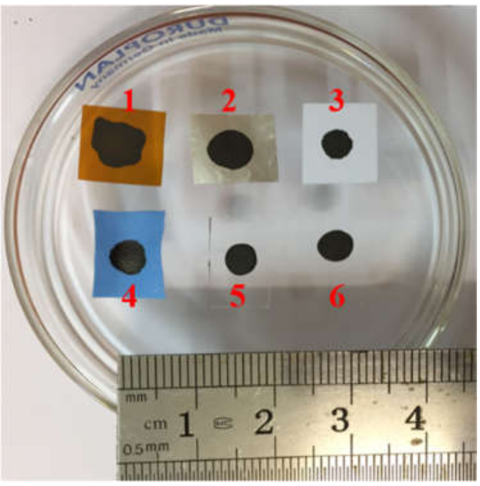

Supplementary Fig. 2 P/G ink can coat on different substrates, including Kapton (**1**), aluminum foil (**2**), paper (**3**), nitrile rubber (**4**), polydimethylsiloxane (PDMS) (**5**), and glass (**6**).

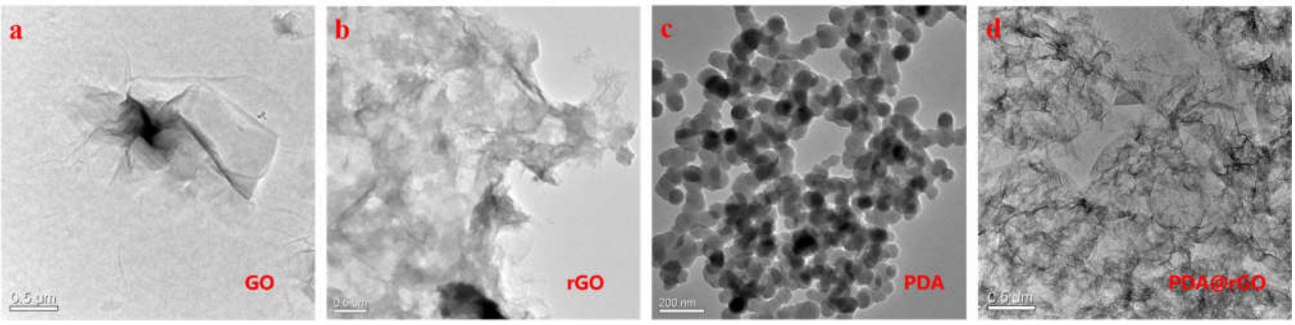

Supplementary Fig. 3 TEM images of GO (**a**), rGO (**b**), PDA (**c**), and PDA@rGO (**d**).

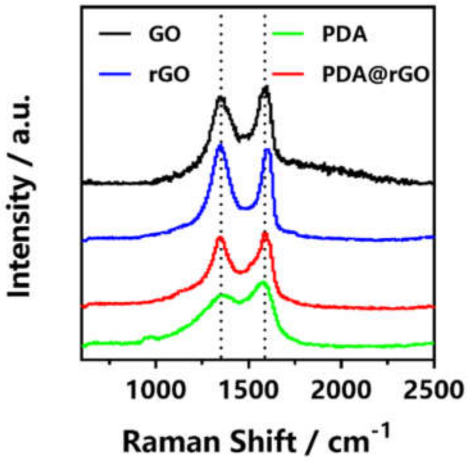

I_D/I_G (cm^{-1})	GO	rGO	PDA	PDA@rGO
I_{1352}/I_{1588}	0.9138	1.1036	0.3621	0.9892

Supplementary Fig. 4 Raman spectra of GO, rGO, PDA, and PDA@rGO. The increase in I_D/I_G of rGO and PDA@rGO proved the reduction of GO.

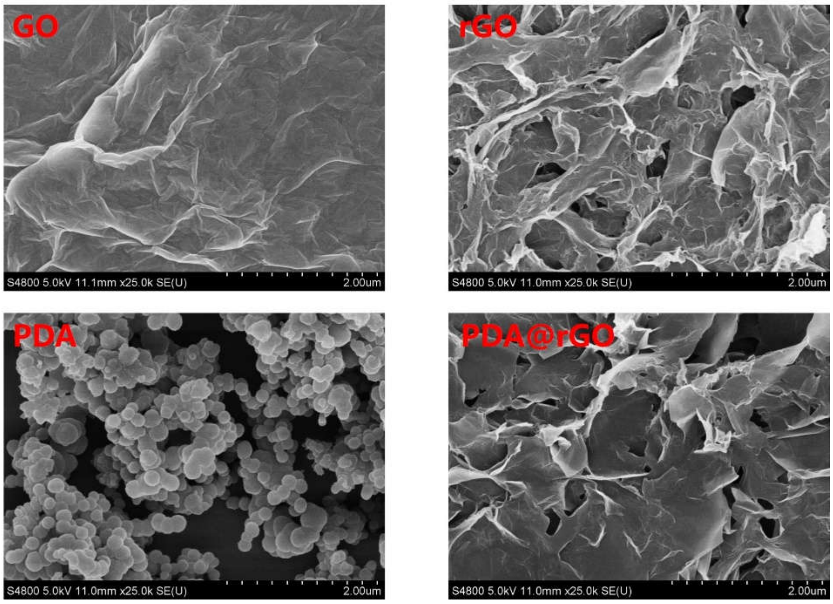

Supplementary Fig. 5 SEM images of GO (**a**), rGO (**b**), PDA (**c**), and PDA@rGO (**d**).

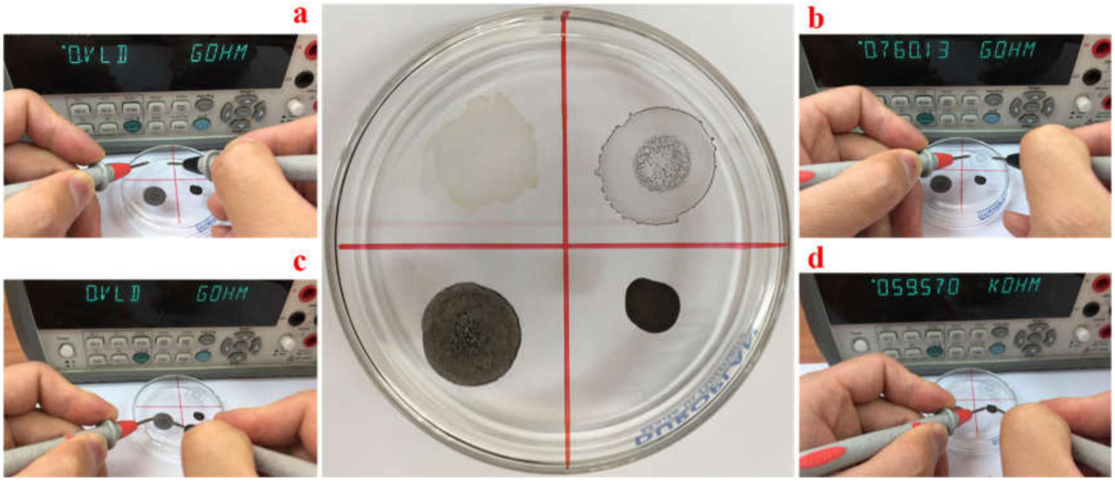

Supplementary Fig. 6 Images show the deposited droplets of GO, rGO, PDA and PDA@rGO on glass, and the measurement of their resistances.

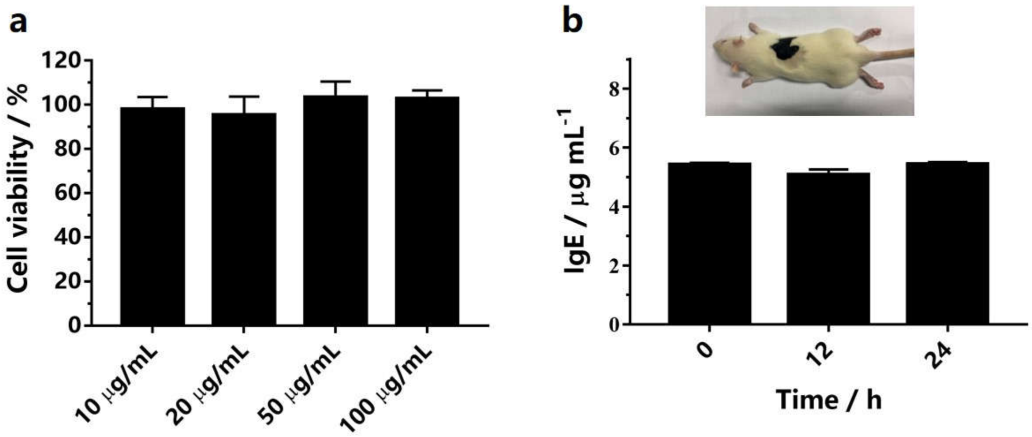

Supplementary Fig. 7 (a) Cell viability of 293 cells determined from WST-8 assay after exposure to different concentrations of PDA@rGO for 24 h. WSK-8, which would not react with rGO, was employed to measure the cytotoxicity of the as-prepared PDA@rGO. Data are presented as the mean ± SD. (**b**) Time-course level of immunoglobulin E (IgE, one of immune markers) in blood of young normal Sprague-Dawley rats, whose part of (shaved) skin was covered with P/G ink. Blood samples (0.8 mL) were taken every 12h and the levels of IgG were measured using a commercially available ELSA kit. Data are presented as the mean ± SD.

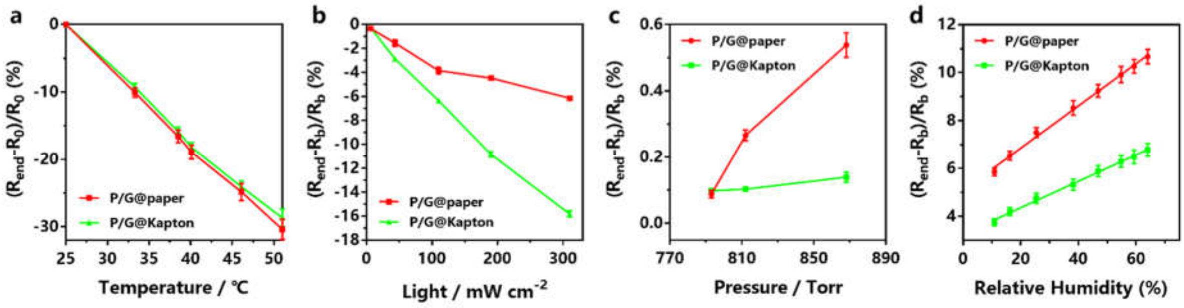

Supplementary Fig. 8 The responses of P/G@paper and P/G@Kapton sensors to temperature (**a**), light exposure (**b**), air pressure (**c**), and relative humidity (**d**). Data are presented as the mean ± SD.

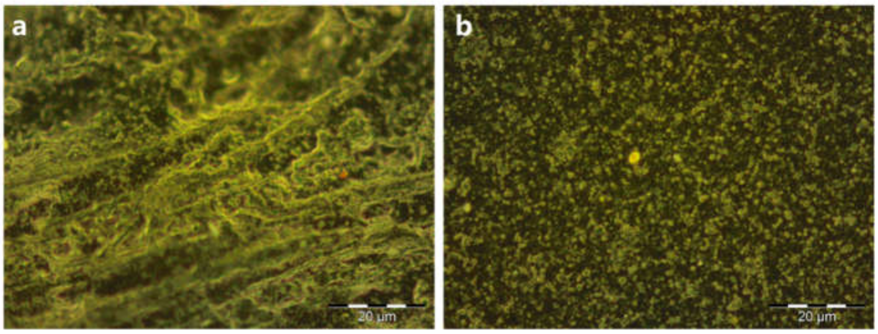

Supplementary Fig. 9 Microscope images of P/G@paper (**a**) and P/G@Kapton (**b**).

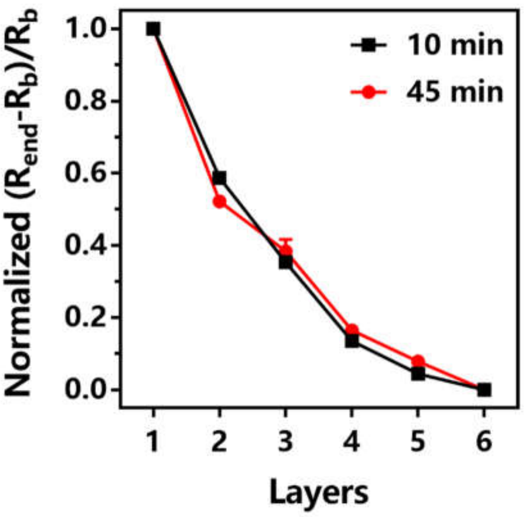

Supplementary Fig. 10 Investigation of the effect of air exposure time (10 min and 45 min) on the OHSA's features of layer-related resistance changes. Data are presented as the mean ± SD.

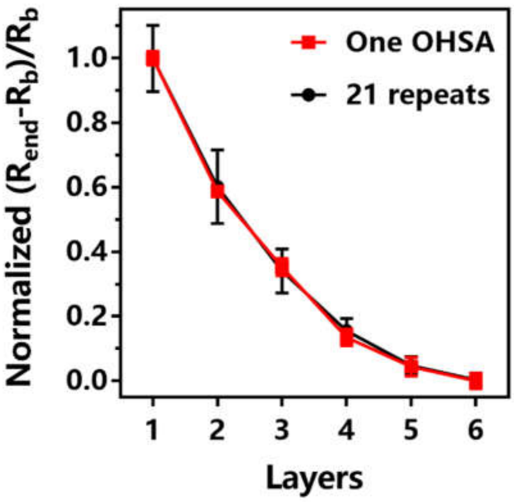

Layers	1	2	3	4	5	6
RSD (%)	10.22	11.42	6.84	3.80	2.83	2.15

Supplementary Fig. 11 Investigation of the reproducibility of parallel-made one-sided OHSAs regarding their features of layer-involved resistance changes. Data are presented as the mean ± SD.

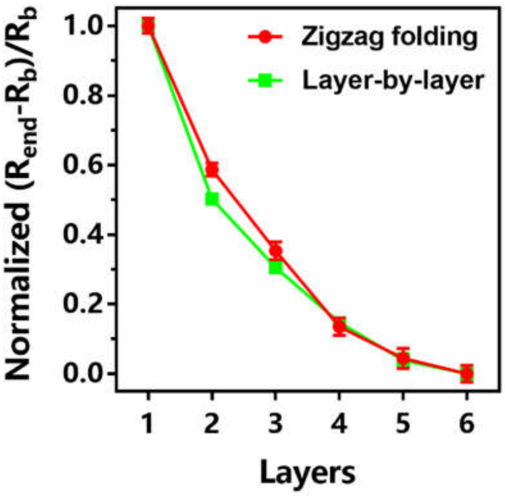

Supplementary Fig. 12 Comparison of the features of layer-involved resistance changes in zigzag folding OHSA and layer-by-layer OHSA. Data are presented as the mean ± SD.

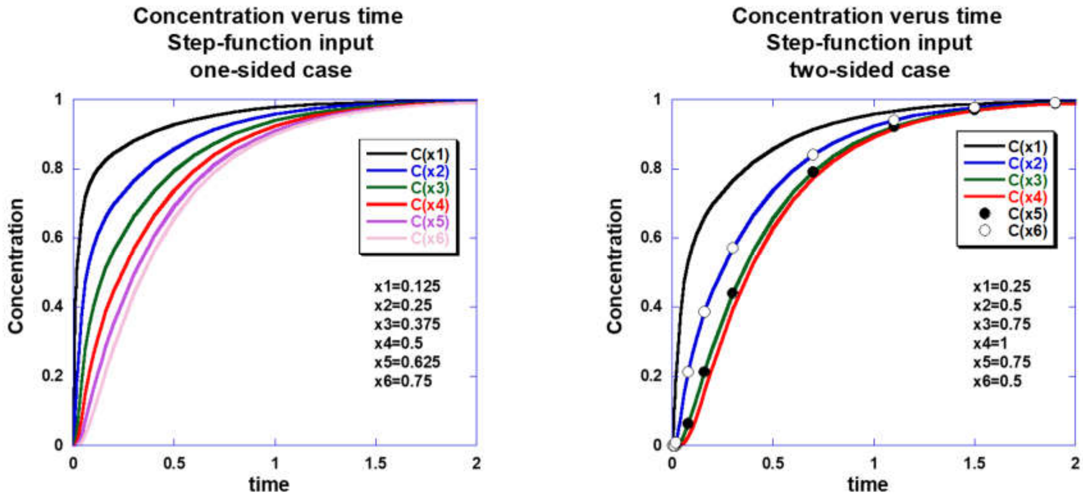

Supplementary Fig. 13 Plots of concentration versus time step-function for the six (dimensionless) sensor positions within the one-sided and two-sided OHSAs (porous block of material).

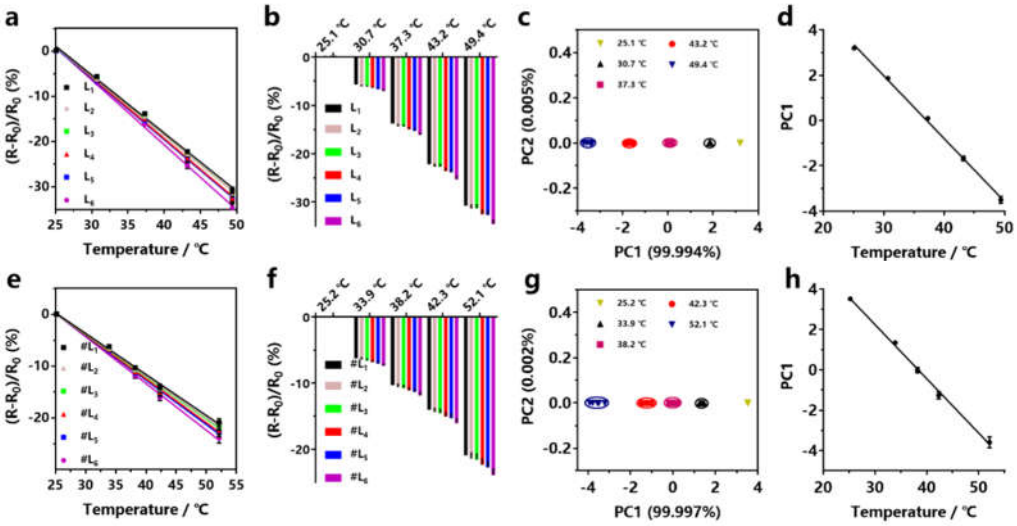

Supplementary Fig. 14 The sensing performances of origami hierarchical sensor array (OHSA) to temperature. Plots of different layers in two-sided OHSA (**a**) and one-side OHSA (**e**) response to temperatures measured under constant humidity and light exposure. Data are presented as the mean ± SD. (R-R$_0$)/R$_0$-response pattern of two-sided OHSA (**b**) and one-side OHSA (**f**) against temperature. Data are presented as the mean ± SD. 2D canonical score plot for the (R-R$_0$)/R$_0$-response patterns of two-sided OHSA (**c**) and one-side OHSA (**g**) as obtained from PCA against temperature. Plots of the first discriminant factor (PC1) of two-sided OHSA (**d**) and one-side OHSA (**h**) vs temperature. Data are presented as the mean ± SD.

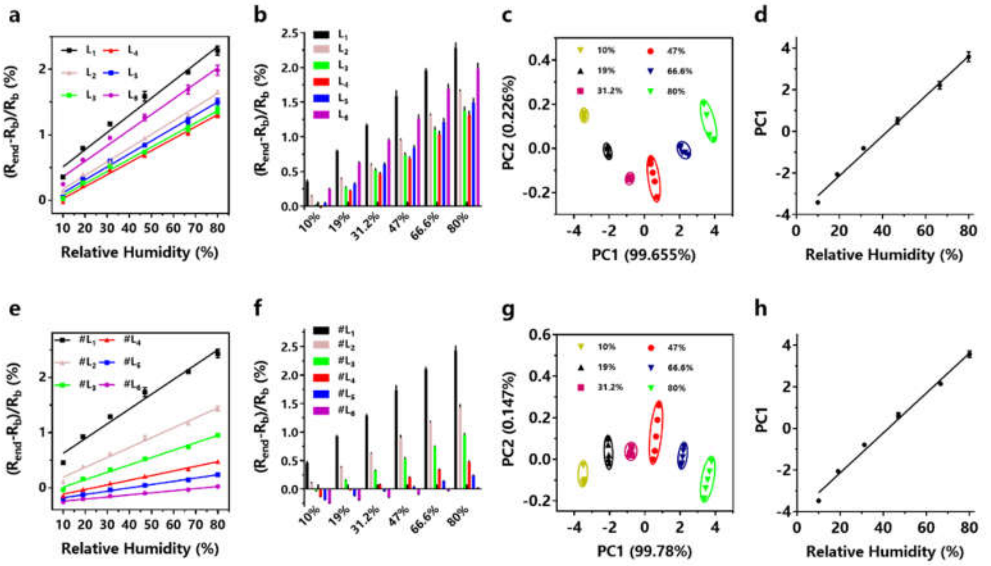

Supplementary Fig. 15 The sensing performances of origami hierarchical sensor array (OHSA) to relative humidity. Plots of different layers in two-sided OHSA (**a**) and one-side OHSA (**e**) response to relative humidity measured under constant temperature and light exposure. Data are presented as the mean ± SD. (R-R$_0$)/R$_0$-response pattern of two-sided OHSA (**b**) and one-side OHSA (**f**) against relative humidity. Data are presented as the mean ± SD. 2D canonical score plot for the (R-R$_0$)/R$_0$-response patterns of two-sided OHSA (**c**) and one-side OHSA (**g**) as obtained from PCA against relative humidity. Plots of the first discriminant factor (PC1) of two-sided OHSA (**d**) and one-side OHSA (**h**) vs relative humidity. Data are presented as the mean ± SD.

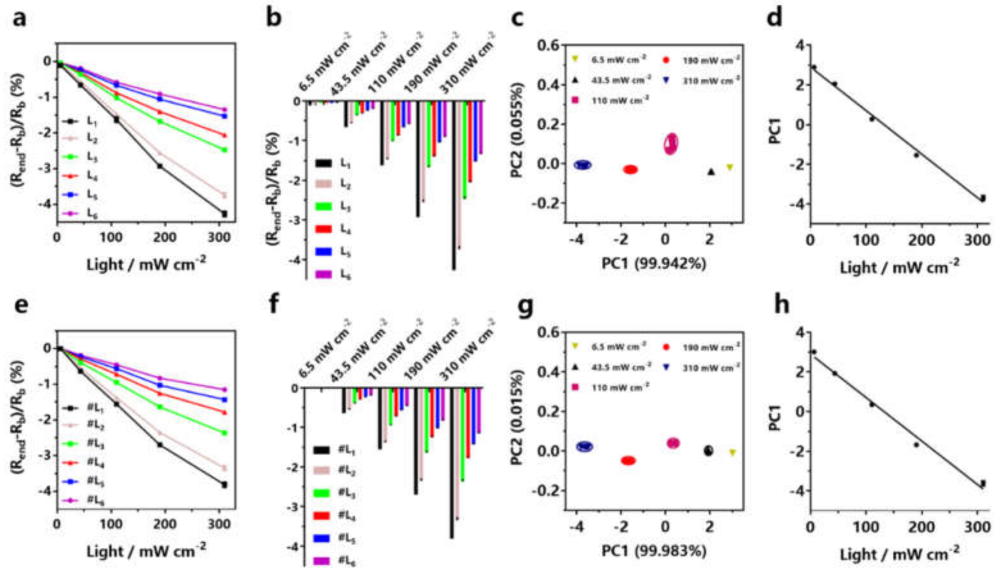

Supplementary Fig. 16 The sensing performances of origami hierarchical sensor array (OHSA) to light exposure. Plots of different layers in two-sided OHSA (**a**) and one-side OHSA (**e**) response to light exposure measured under constant temperature and relative humidity. Data are presented as the mean ± SD. $(R-R_0)/R_0$-response pattern of two-sided OHSA (**b**) and one-side OHSA (**f**) against light exposure. Data are presented as the mean ± SD. 2D canonical score plot for the $(R-R_0)/R_0$-response patterns of two-sided OHSA (**c**) and one-side OHSA (**g**) as obtained from PCA against light exposure. Plots of the first discriminant factor (PC1) of two-sided OHSA (**d**) and one-side OHSA (**h**) vs light exposure. Data are presented as the mean ± SD.

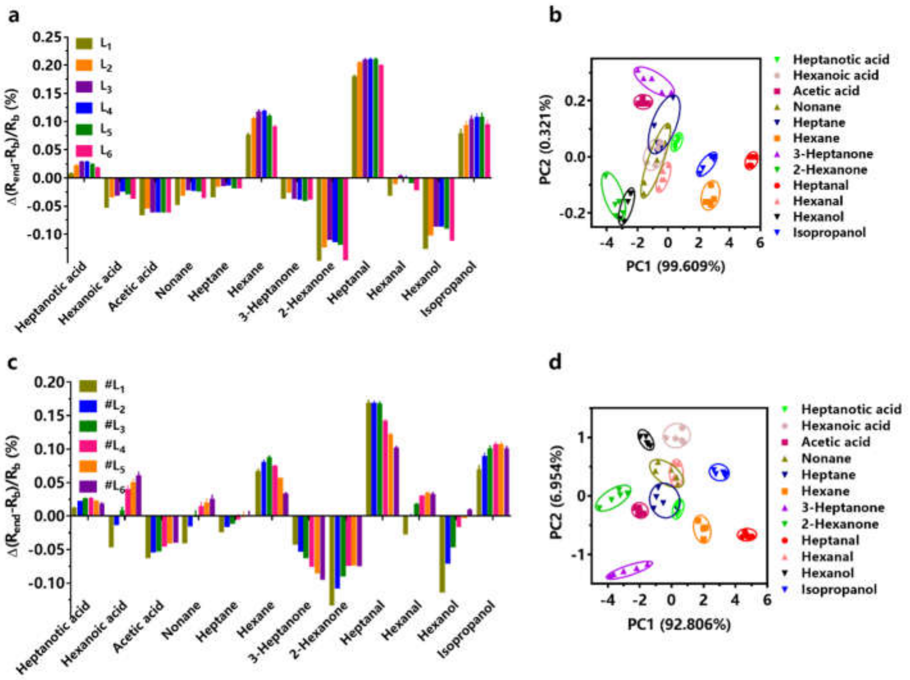

Supplementary Fig. 17 The sensing performances of origami hierarchical sensor array (OHSA) to VOCs. $\Delta[(R_{end}-R_b)/R_b]$-response pattern of two-sided OHSA (**a**) and one-sided OHSA (**c**) against 10 ppm VOCs. Data are presented as the mean ± SD. 2D canonical score plot for the $(R_{end}-R_b)/R_b$-response pattern of two-sided OHSA (**b**) and one-sided OHSA (**d**) as obtained from PCA against VOCs.

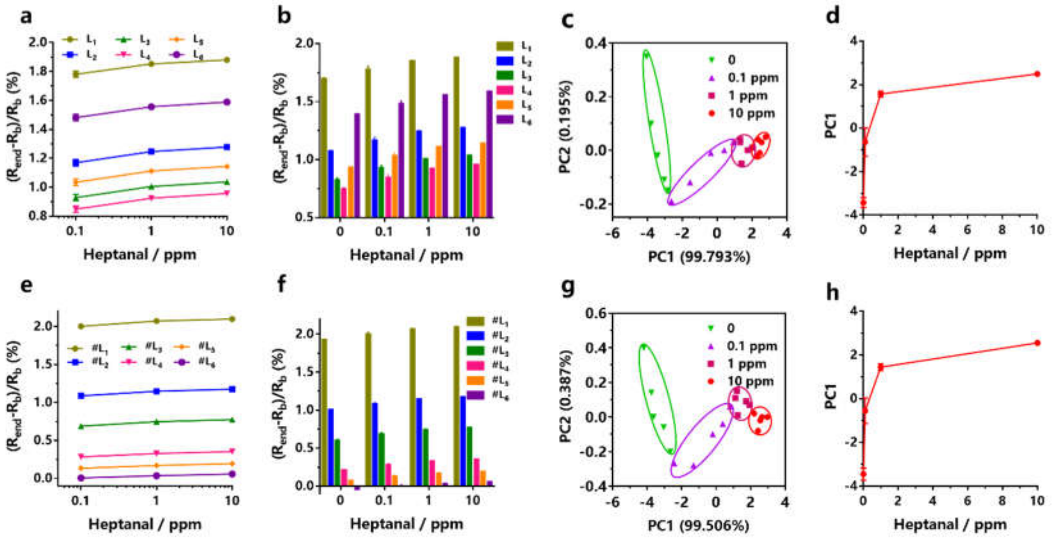

Supplementary Fig. 18 Plots of different layers of two-sided OHSA (**a**) and one-sided OHSA (**e**) responses to Heptanal from 0 to 10 ppm. Data are presented as the mean ± SD. $(R_{end}-R_b)/R_b$-response pattern of two-sided OHSA (**b**) and one-sided OHSA (**f**) against Heptanal from 0 to 10 ppm. Data are presented as the mean ± SD. 2D canonical score plot for the $(R_{end}-R_b)/R_b$-response pattern of two-sided OHSA (**c**) and one-sided OHSA (**g**) as obtained from PCA against Heptanal from 0 to 10 ppm. Plots of the first discriminant factor (PC1) of two-sided OHSA (**d**) and one-sided OHSA (**h**) vs Heptanal. Data are presented as the mean ± SD.

Supplementary Table 1. Name, molecular weight and chemical formula of the selected VOCs.

Type	Name	Mw	Formula
**Acids**	Heptanoic acid	130.19	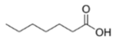
	Hexanoic acid	116.16	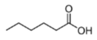
	Acetic acid	60.05	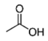
**Alkanes**	Nonane	128.26	
	Heptane	100.2	
	Hexane	86.18	
**Ketones**	3-Heptanone	114.19	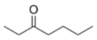
	2-Hexanone	100.16	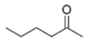
**Aldehydes**	Heptanal	114.18	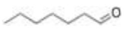
	Hexanal	100.16	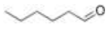
**Alcohols**	Hexanol	102.17	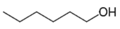
	Isopropanol	60.1	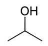
**Xylene isomers**	*o*-Xylene	106.16	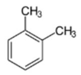
	m-Xylene	106.16	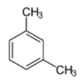
	p-Xylene	106.16	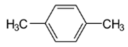
**Octanol enantiomers**	(*R*)-(−)-2-Octanol	130.23	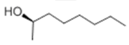
	(*S*)-(+)-2-Octanol	130.23	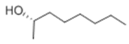
**Butanol enantiomers**	(*R*)-(−)-2-Butanol	74.12	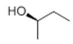
	(*S*)-(+)-2-Butanol	74.12	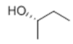

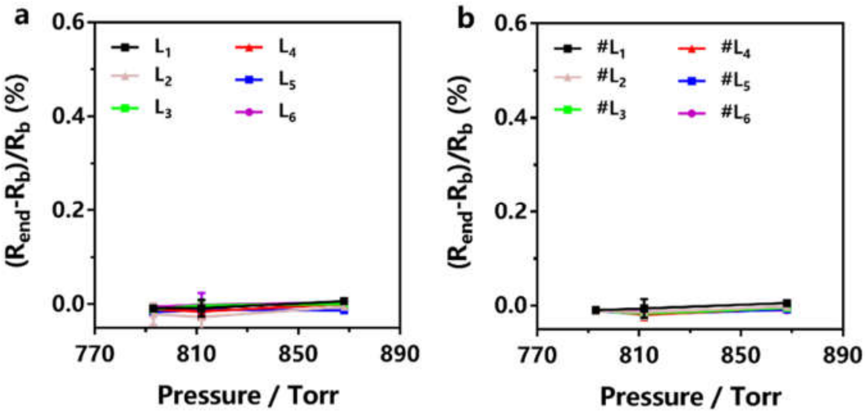

Supplementary Fig. 19 Plots of different layers in two-sided OHSA (**a**) and one-sided OHSA (**b**) in a stainless-steel chamber response to air pressure from 793 to 868 Torr controlled by gas generator under constant humidity and temperature. Data are presented as the mean ± SD.

Supplementary Table 2. Summarization of the layer-dependent response sensitivity of two-sided OHSA and one-sided OHSA toward temperature, RH, light and VOC (Heptanal as a model).

Sensor	Stimulus	Response sensitivity (% per stimulus*)					
		Layer 1	Layer 2	Layer 3	Layer 4	Layer 5	Layer 6
Two-sided OHSA	Temperature	1.272	1.295	1.294	1.343	1.347	1.427
	RH	0.0276	0.0216	0.0193	0.0191	0.0207	0.0248
	Light	0.0137	0.0121	0.008	0.0066	0.0049	0.0043
	Heptanal	0.0179	0.0204	0.0209	0.021	0.021	0.0199
One-sided OHSA	Temperature	0.7793	0.7966	0.8035	0.8321	0.8472	0.8909
	RH	0.0282	0.0189	0.014	0.088	0.0062	0.004
	Light	0.0126	0.011	0.0078	0.0059	0.0047	0.0038
	Heptanal	0.0169	0.0169	0.0167	0.0141	0.0121	0.0101
1-decanethiol-capped Au-MCNPs	Temperature	0.65		Ref: *Adv. Mater. Technol.* 2017, 2, 1600206			
	RH	0.54					
Photoresistor	Light	14.57		Commercially available CdS photoconductive cells (GL5528)			
Salicylic acid-doped polyaniline thin film	Hexanal	~0.03		Ref: *Adv. Funct. Mater.* 2017, 1703147			

*Temperature: per °C; RH: per RH; Light: per mW cm^{-2}; Heptanal or Hexanal: per ppm

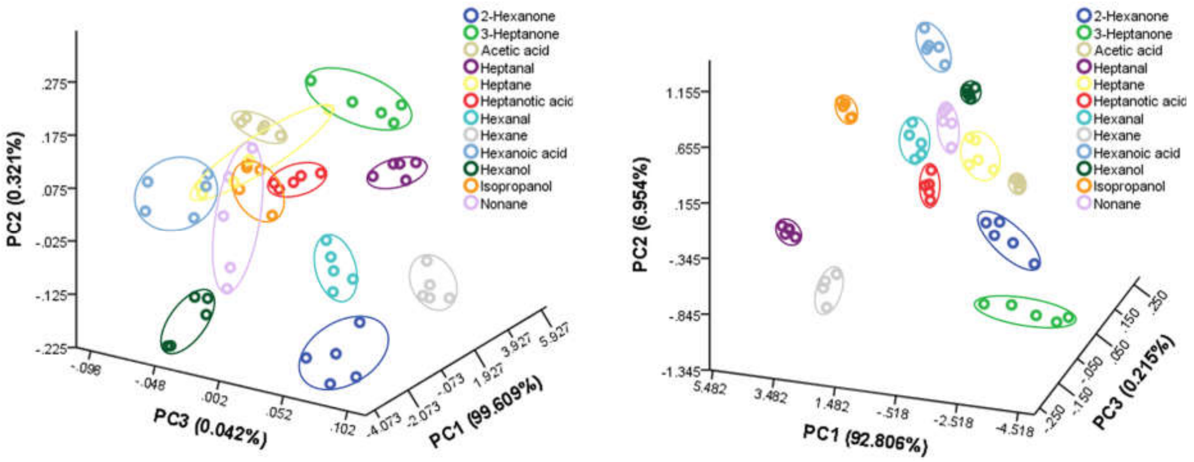

Supplementary Fig. 20 3D canonical score plot for the $(R_{end}-R_b)/R_b$-response pattern of two-sided OHSA (**a**) and one-sided OHSA (**b**) as obtained from PCA against VOCs.

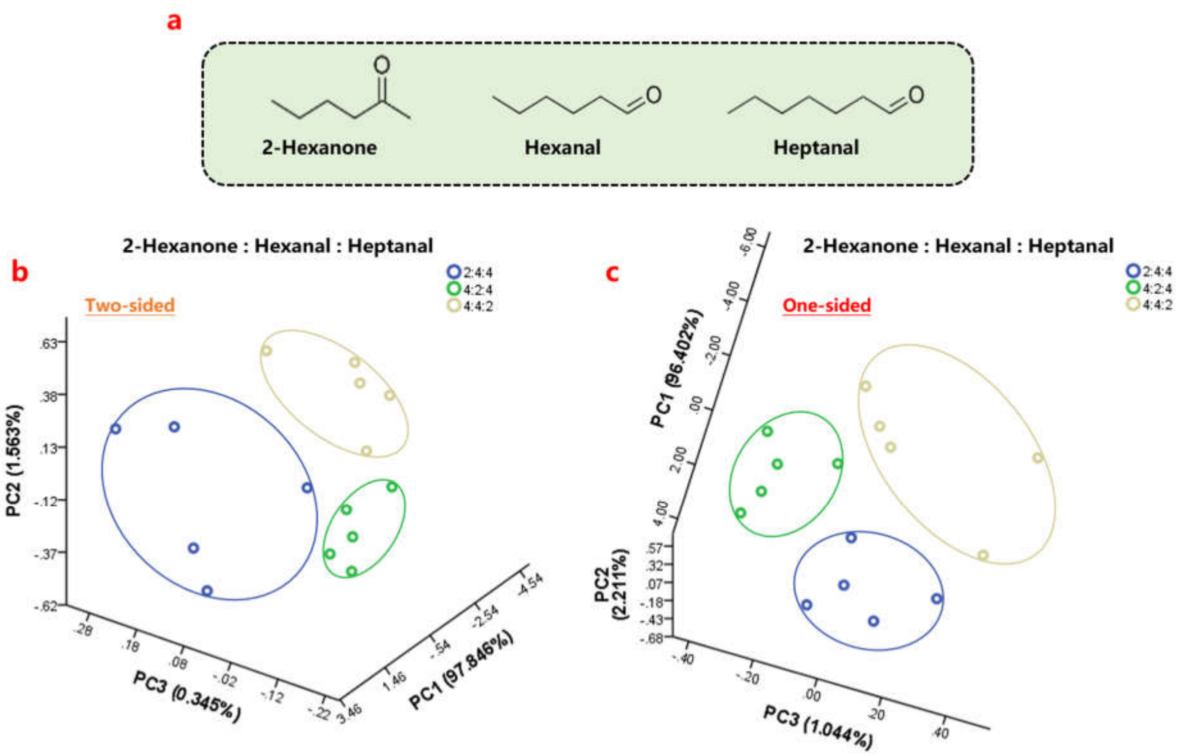

Supplementary Fig. 21 (**a**) Structural formula of 2-Hexanone, Hexanal, and Heptanal. 3D canonical score plots for two-sided OHSA (**b**) and one-sided OHSA (**c**) against the mixtures of 2-Hexanone, Hexanal, and Heptanal at different mass ratios indicated (ppm), respectively.

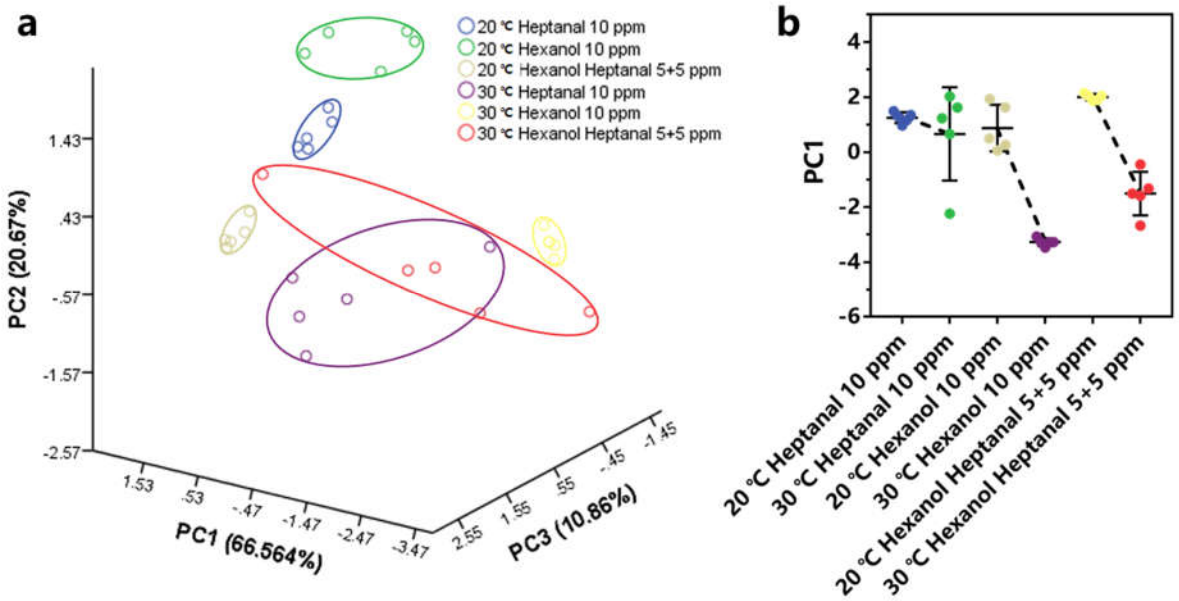

Supplementary Fig. 22 (**a**) 3D canonical score plot for one-sided OHSA against VOCs (10 ppm Hexanol, 10 ppm Heptanal and the mixture of 5 ppm Hexanol + 5 ppm Heptanal) at varied temperature conditions (20 °C and 30 °C), respectively. (**b**) Plot of the first discriminant factor (PC1) for one-sided OHSA vs the pattern of VOCs indicated at varied temperature conditions (20 °C and 30 °C). Data are presented as the mean ± SD.

Supplementary Table 3. The optimized structures of three rGO-PDA-Xylene complexes and their HOMO-LUMO levels. The modeling is done by using APFD/6-31G*.

	rGO-PDA-m-Xylene	rGO-PDA-o-Xylene	rGO-PDA-p-Xylene
	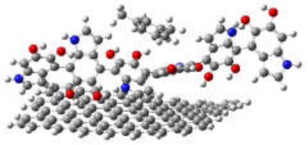	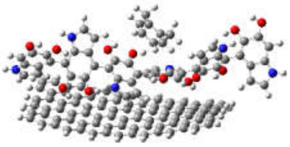	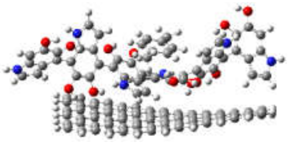
Lumo [eV]	**-3.79**	**-3.73**	**-3.76**
Homo [eV]	**-4.08**	**-4.01**	**-4.05**

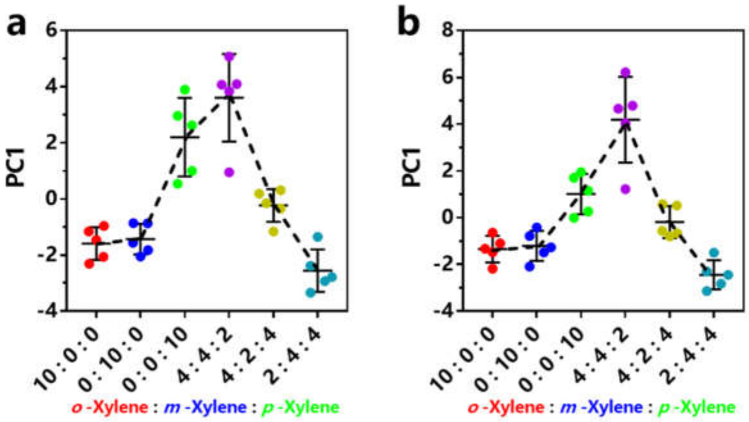

Supplementary Fig. 23 Plot of the first discriminant factor (PC1) for two-sided OHSA (**a**) and one-sided OHSA (**b**) vs the pattern of Xylene isomers (10 ppm) and their mixtures at the different mass ratios. Data are presented as the mean ± SD.

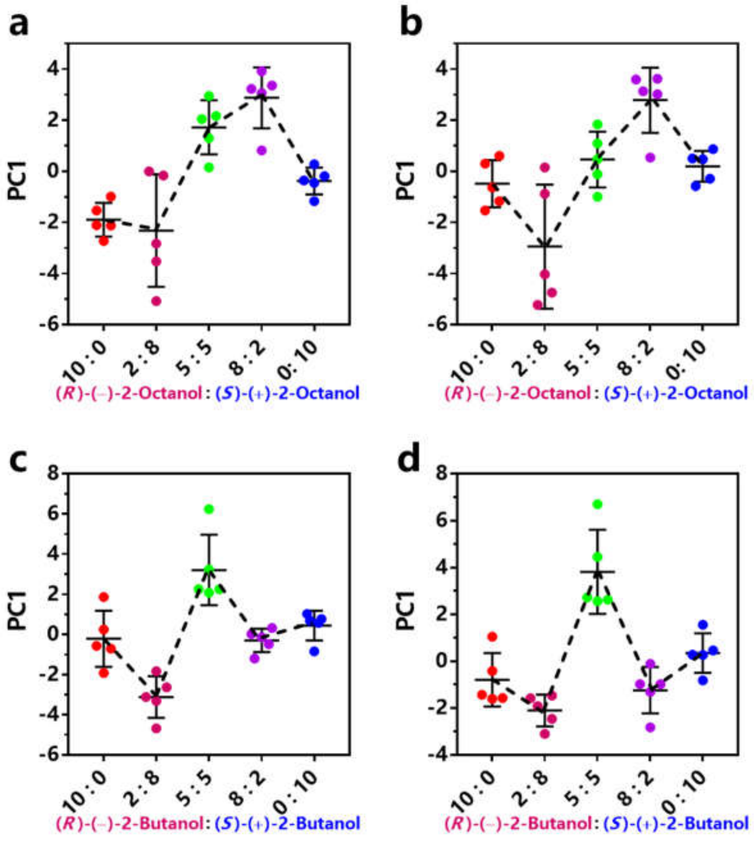

Supplementary Fig. 24 Plot of the first discriminant factor (PC1) for two-sided OHSA (**a, b**) and one-sided OHSA (**c, d**) vs the pattern of 2-Octanol enantiomers (10 ppm) and 2-Butanol enantiomers (10 ppm) and their mixtures at different mass ratios indicated, respectively. Data are presented as the mean ± SD.

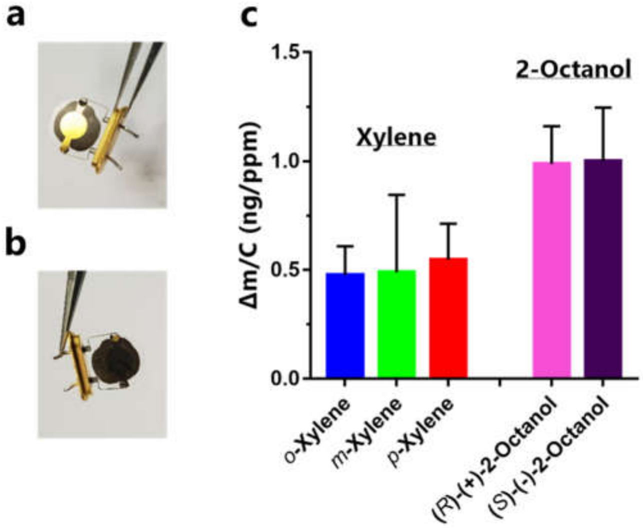

Supplementary Fig. 25 Images of gold-plated QCM resonator (5 mm in diameter) (**a**) and P/G ink-coated gold-plated QCM resonator (**b**). (**c**) Bar represent the mass changes of the P/G ink-coated gold-plated QCM resonator caused by exposures to Xylene isomers and 2-Octanol enantiomers. Data are presented as the mean ± SD.

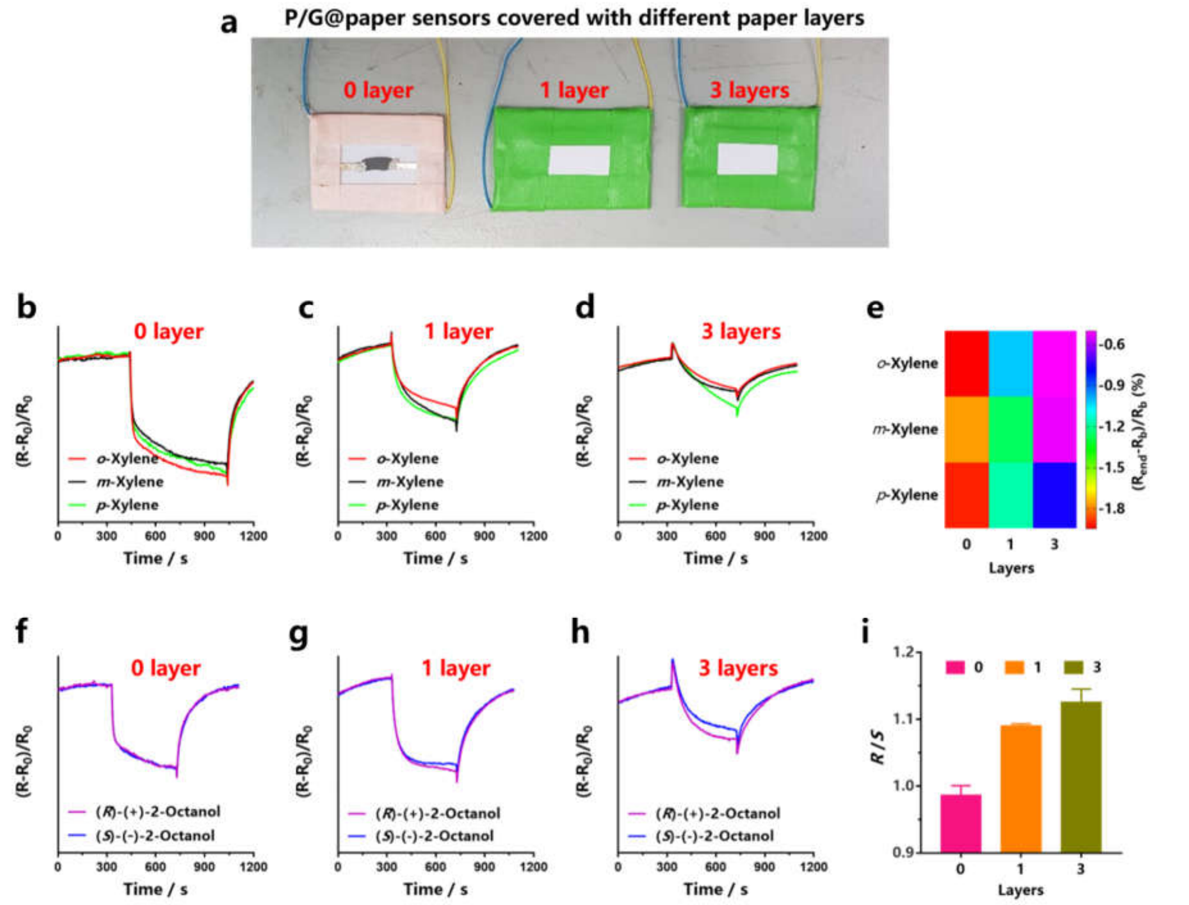

Supplementary Fig. 26 (**a**) Configuration of P/G@paper sensors covered with different paper layers.

Relative resistance changes of P/G@paper sensors covered with different paper layers toward Xylene isomers (**b-d**) and 2-Octanol enantiomers (**f-h**). (**e**) Heap map of the (R_{end}-R_b)/R_b-responses of P/G@paper sensors covered with different paper layers toward Xylene isomers. (**i**) Bar represent the chiral discrimination of P/G@paper sensors covered with different paper layers toward 2-Octanol enantiomers. Note: *R/S* means the ratio of the (R_{end}-R_b)/R_b-responses between 2-Octanol enantiomers. Data are presented as the mean ± SD.

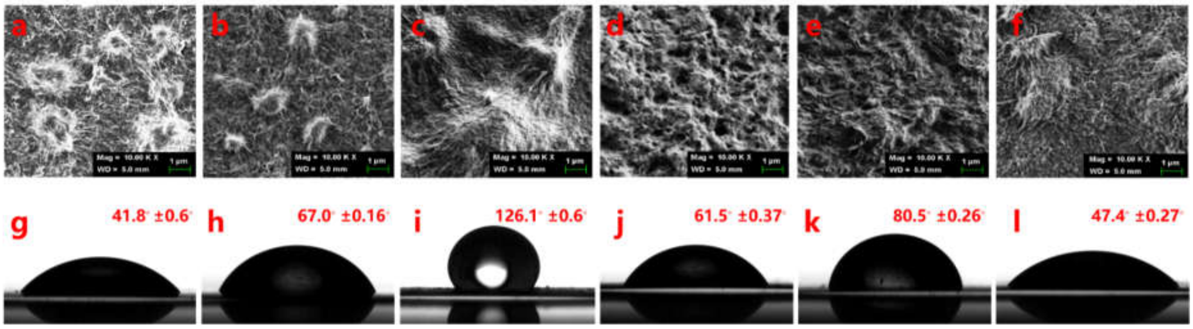

Supplementary Fig. 27 Scanning electron microscope (SEM) (**a-f**) and contact angle (**g-l**) images of the surface morphology of deposited inks: P/G ink, ***T_1***-P/G ink, ***T_2***-P/G ink, ***N_1***-P/G ink, ***N_2***-P/G ink, and ***P***-P/G ink, respectively.

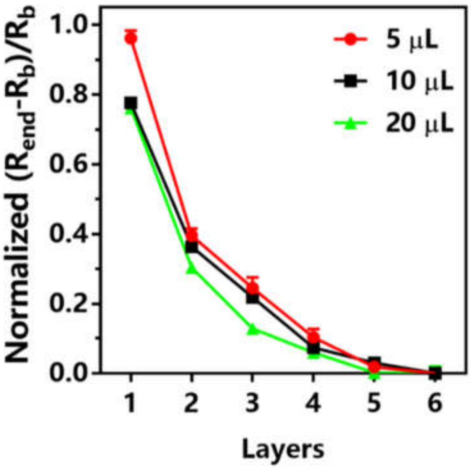

Supplementary Fig. 28 Plots represent the (R_{end}-R_b)/R_b-responses of one-sided OHSA loaded with various amounts of ink toward air exposure, respectively. Data are presented as the mean ± SD.

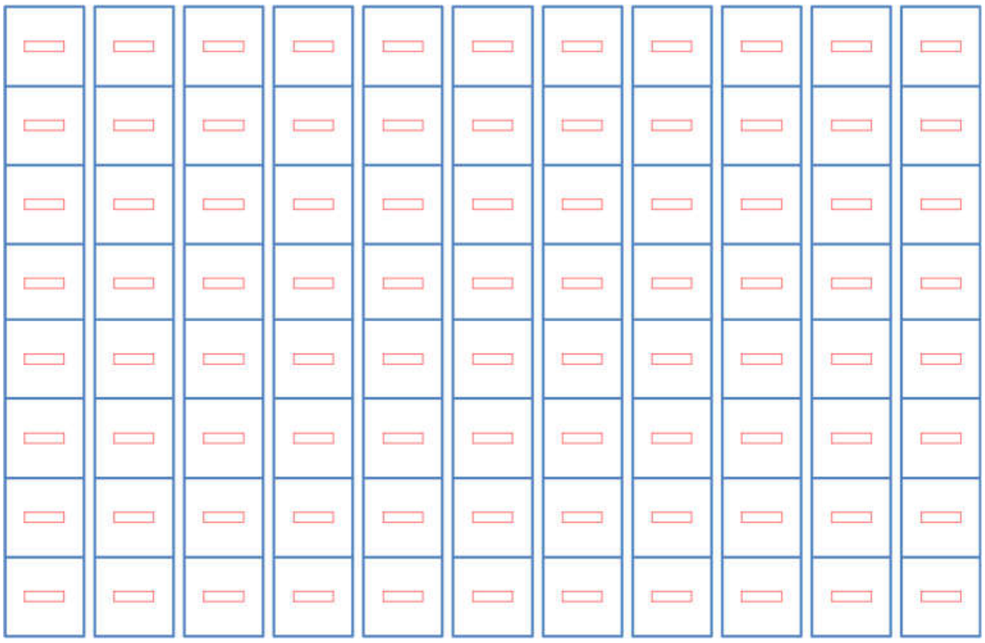

Supplementary Fig. 29 A template for origami designed on PowerPoint with seven detached columns with a size of 2 cm×8 cm. Each column has eight squares with a size of 2 cm×2 cm, and there is one strip with a size of 0.5 cm×1 cm in the middle of each square.
