# Supplementary material for: Time-space-resolved origami hierarchical electronics for ultrasensitive detection of physical and chemical stimuli
Source: Nat Commun. 2019 Mar 8;10:1120. doi: 10.1038/s41467-019-09070-8 (PMC6408588; doi:10.1038/s41467-019-09070-8)
Supplement: Supplementary file 3 — Description of Additional Supplementary Files [file 41467_2019_9070_MOESM3_ESM.docx]

**Description of Additional Supplementary Files**

File Name: Supplementary Movie 1

Description: The Supplementary Movie shows that P/G ink written on skin can act as a water-proof tattoo
