# Supplementary material for: Time-space-resolved origami hierarchical electronics for ultrasensitive detection of physical and chemical stimuli
Source: Nat Commun. 2019 Mar 8;10:1120. doi: 10.1038/s41467-019-09070-8 (PMC6408588; doi:10.1038/s41467-019-09070-8)
Supplement: Supplementary file 2 — Peer Review [file 41467_2019_9070_MOESM2_ESM.pdf]

Reviewers' comments:

Reviewer #1 (Remarks to the Author):

This manuscript reports an origami hierarchical sensor array with a time-space-resolved chromatography-mimic property. The sensor can detect complex physical and chemical stimuli and it also has a potential to distinguish different isomer and chiral enantiomers. Although the concept would be novel, the paper has some quantitative ambiguity. I recommend further consideration to clear up the points before the manuscript is accepted.

1. How about resistivity of the flexible resistor made from the P/G ink? What is the initial resistance R0 in Figure 1j? Please clarify the absolute values of resistances.

2. In Fig. 1k-n, if multiple parameters change at the same time, how can we determine each parameter from the resistance of the sensor? e.g., temperature and relative humidity usually changes simultaneously.
3. It is important to discuss the response/recovery time and the sensitivity on the sensors. Please compare the properties with previous reported paper-drawn sensors.

4. In Supplementary Fig. 5, are there any cracks in each film? Please add SEM images of the films.
5. Why do VOC gas molecules change the resistance of the sensor? Please explain the mechanism, including why isomer and enantiomer can be distinguished by this mechanism.

6. Is the P/G ink painted directly on skin and fingernail in the supporting video? If so, please comment on the safety of the ink to human health. Why is the P/G ink waterproof? Since the P/G ink is dispersed in ethanol, the dried ink seems to be also soluble in water. If the P/G ink is not soluble in water, how can we remove the ink from skin?

Reviewer #2 (Remarks to the Author):

1- The idea of applying origami concept to bring a 3D structure is novel, however the fundamental principles leading the transduction mechanism of the sensor work presented here has been conducted for decades. The use of PCA method to discriminate pure chemical mixtures is not novel and does not have demonstrated practical applications in any current commercial device for the intended purpose reported in this manuscript. Furthermore, the sensor array reports changes to multiple chemical and physical stimuli such as temperature, humidity, and pressure. Controlling all these physical variables together with the chemical variables in a real sensing scenario is difficult.

2- Additionally, the features of the system could be subjected to the way the sensors are packed, and reproducibility may be an issue if the space between layers is different.

3- Mass transport of the processes leading to a response should be discussed. Passive diffusion vs. different convective conditions may offer differences in the response discrimination patterns.

4- On the other hand, the comparison of time-space resolve chromatography-mimic is misleading because there is no discrimination of different chemicals based on chemical interactions that accelerate or promote the mass transport in a structure to induce responses capture at different times. The term: "time-space-resolved chromatography-mimic OHSA" should be replaced by a name reflecting the actual system, example: "high discrimination PCA OHSA"

5- There are several exaggerative sentences that should be completely eliminated or replaced given the system does no necessary applies to the specific mentioned applications. Examples are:

*Therefore, our time-space-resolved chromatography-mimic OHSA could reach help reach a future goal for non-invasive medical diagnostics based on the analysis of VOCs emitted from "diverse bodily sources".

*It should provide a potential tool for profiling and deciphering homologous VOCs (e.g. enantiomers) toward "precision medicine".

Because of these intriguing features, the OHSA-based hierarchical design presented herein opens the door for the creation of smart time-space-resolved chromatography-mimic electronics, and this would boost more versatile and meaningful intelli-sense applications toward precise medicine, healthcare monitoring, etc.

Reviewer #3 (Remarks to the Author):

The manuscript describes the low-cost fabrication and properties of PDA-rGO based origami sensor array for the detection of different physical (temperature, humidity, pressure, light) and chemical (VOC) stimuli. One of the uniqueness of this sensor is its ability to identify structural isomers and chiral recognition of enantiomers. Although the scientific content is exciting to read, the grammar and English should be improved considerably. Additionally,

1. In section 'P/G ink-loaded origami hierarchical sensor array (OHSA)' authors should consider identifying the type of paper used for the origami sensor and the thickness/amount of the ink deposited on the substrate. It would improve the manuscript if the authors commented on the effect of thickness of ink on the adhesion on various substrates? Do paper type and paper quality affect the resistivity readings?

2. What is the meaning of 'vacuum clean'? Was there a sub-atmospheric pressure in a stainless steel chamber to remove 'air species'? As per figure 8a and b in the supplemental materials, the pressure in the chamber was varied from 793 to 868 Torr- these pressures are not associated with the generally accepted definition of the term vacuum.

3. Figure 2g and h: the relative resistivity seems to saturate with time. Did authors observe this saturation? If yes, what are the saturation values for different layers?

4. In section 'Multifunctional monitoring of multiple stimuli by using OHSA' authors state: Notably from Supplementary Fig. 12b and d, it can be seen that most of the tested VOCs ….. were clearly differentiated in the PCA plots using both two-sided OHSA and one-sided OHSA. However in figure 3i and j and in figure 12 b and d, values for acetic acid, heptane, nonane etc. do overlap. Authors need to address this.

5. Since both one sided and two-sided origami arrays can be used to detect the physical and chemical stimuli, authors should discuss advantages of using one sided and two sided OHSA over each other.

6. What will be the response of OHSA if instead of zigzag folding, each individual layer is cut at the fold and stacked on top of each other?

Actions (revisions) appear in red; in the marked-up revised version of the manuscript. Those changes are highlighted. Text from the original or revised manuscript appears in *italics*.

REVIEWER 1		
	**Remarks to the Author**	**Response to the Reviewer**
**General comments:**		
	This manuscript reports an origami hierarchical sensor array with a time-space-resolved chromatography-mimic property. The sensor can detect complex physical and chemical stimuli and it also has a potential to distinguish different isomer and chiral enantiomers. Although the concept would be novel, the paper has some quantitative ambiguity. I recommend further consideration to clear up the points before the manuscript is accepted.	We thank for your positive comments and appreciate your feedback. In the revised manuscript, we have carefully addressed your concerns and improved the quality of our work.
**Specific comments:**		
1.	How about resistivity of the flexible resistor made from the P/G ink? What is the initial resistance R_0 in Figure 1j? Please clarify the absolute values of resistances.	The sheet resistivity of P/G ink was measured by a four-point probe nanovoltmeter. Its value was found to be 1825.08 S/m.<br><br>This point as well as the absolute values of resistances of P/G ink sensors are now mentioned in the text, via the following text (page 5):<br><br>*"its sheet resistance is measured to be 1825.08 S/m" in Page 4 & "The initial resistance (R_0) of P/G@paper is 45.3 kΩ and the R_0 of P/G@Kapton is 77.3 kΩ"*
2.	In Fig. 1k-n, if multiple parameters change at the same time, how can we determine each parameter from the resistance of the sensor? e.g., temperature and relative humidity usually changes simultaneously.	We have now examined the plausibility of P/G@paper and P/G@Kapton sensors to discriminate between various physical and chemical parameters in a complex environment / mixture. We found that the use of individual sensor cannot reach the anticipated separation (Fig. 1). On the other hand, we have found that the use of P/G ink-loaded origami hierarchical sensors array (OHSA) as a smart ‘e-nose/e-tongue’, side by side with a set of calibration curves, would be good enough to reach the targeted discrimination between the various parameters in the complex environment. As a

		proof-of-concept, we have examined the plausibility to discriminate between VOCs (10 ppm Hexanol, 10 ppm Heptanal and 5 ppm Hexanol+5 ppm Heptanal mixture) at varied temperature conditions (20°C and 30°C) using our OHSA, respectively (Supplementary Fig. 22). The 3D PCA plot in Supplementary Fig. 22a proves that the canonical resistance-response patterns from aforesaid VOCs can be well differentiated using our one-sided OHSA under both 20°C and 30°C. Since the second discriminant factor (PC2) and the third discriminant factor (PC3) of the 3D PCA plots were <40%[13] (Supplementary Fig. 22a), it was reasonable and acceptable to adopt the first discriminant factor (PC1) to depict the pattern of aforesaid VOC samples under different temperatures (Supplementary Fig. 22b). These VOC samples at different temperature conditions can be mapped for tracing each parameter alone, verifying the robustness of our OHSA for the usage in complex environment.<br><br>We have now clarified this aspect in the text by adding (Page 13):<br><br>*"Moreover, considering that many stimuli usually change simultaneously, we have examined the possibility to discriminate between various parameters existing in a specific / given complex environment. As a proof-of-concept, we have performed the discrimination of VOCs (10 ppm Hexanol, 10 ppm Heptanal and the mixture of 5 ppm Hexanol + 5 ppm Heptanal) at varied temperature conditions (20 ℃ and 30 ℃) using our OHSA, respectively (Supplementary Fig. 22). The 3D PCA plot in Supplementary Fig. 22a proves that the canonical resistance-response patterns from aforesaid VOCs can be well respectively differentiated using our one-sided OHSA under both 20 ℃ and 30 ℃. Since the second discriminant factor (PC2) and the third discriminant factor (PC3) of the 3D PCA plots were <40%[13] (Supplementary Fig. 22a), it was reasonable and acceptable to adopt the first discriminant factor (PC1) to depict the pattern of aforesaid VOC samples under different temperatures (Supplementary Fig.*

		22b). From the Supplementary Fig. 22b, these VOC samples in different temperature conditions can be mapped for tracing each parameter alone, verifying the robustness of our OHSA for the usage in complex environment."
3.	It is important to discuss the response/recovery time and the sensitivity on the sensors. Please compare the properties with previous reported paper-drawn sensors.	In the revised manuscript, we have summarized the response/recovery time and the sensitivity of the sensors. We have clarified this aspect in the text by adding (Page 11):<br><br>*"The layer-dependent response sensitivity of both the two OHSAs toward temperature, RH, light and VOC (Heptanal as a model) was also summarized (Table S2)"*<br><br>An updated literature survey shows that there are almost no ink-based paper sensors that have been used for discrimination of multiple parameters. Therefore, the best that could be done is to compare the performance of our sensors with the most relevant sensors (whether paper-based or not) reported so far (Table S2). We have clarified this aspect in the text (Page 11) by adding:<br><br>*"and their analytical performances are compared to some reported sensors. For example, due to the excellent thermal conductivity of rGO as a component, our OHSAs have more sensitivity in the sensing of temperature (~1.272% per °C of two-sided OHSA) than that of 1-decanethiol-capped Au-MCNPs sensor (0.65% per °C)[34]. In addition, they have comparable sensitivity in the monitoring of Heptanal (~0.02% per ppm of two-sided OHSA) in contrast to that of maleic acid-doped polyaniline sensor (~0.03% per ppm)[9]."*
4.	In Supplementary Fig. 5, are there any cracks in each film? Please add SEM images of the films.	In the revised manuscript, we have added the SEM images of the films, as shown in Supplementary Fig. 5. We have clarified this aspect in the text by adding (Page 4):<br><br>*"From the results, we can notice that there are some porous structures in the films of rGO and PDA@rGO (Supplementary Fig. 3 and 5), and*

		PDA@rGO has more smoother surface than rGO due to the coating of PDA."
5.	Why do VOC gas molecules change the resistance of the sensor? Please explain the mechanism, including why isomer and enantiomer can be distinguished by this mechanism.	We have used DFT molecular modeling of P/G upon exposure to VOCs to explain the mechanism of resistance changes of the sensor and how to use them for discrimination of isomers (m-Xylene, o-Xylene, and p-Xylene). Namely, according to the formulation of a P/G ink, PDA is believed to self-polymerize from DA during hydrothermal reduction of GO. The non-covalent interaction between PDA and rGO interaction seems to affect the HOMO-LUMO level of P/G ink under exposure. Indeed, we have used Gaussian software to examine these complexes at APFD/6-31G* level. The output of the calculation is shown in Table S3, showing that the HOMO-LUMO levels (and band gap in some extent) vary differently for different isomers. This could explain the resistance change of the sensor in the experiment with different xylene isomers; as HOMO-LUMO levels of complexes varied with respect to the Fermi level of the metal electrode of the sensor. We have clarified this aspect in the text by adding (Page 14):<br><br>*"We have used DFT molecular modeling of P/G upon exposure to VOCs to explain the mechanism of resistance changes of the sensor and how to use them for discrimination of isomers (m-Xylene, o-Xylene, and p-Xylene). Namely, according to the formulation of a P/G ink, PDA is believed to self-polymerize from DA during hydrothermal reduction of GO. The non-covalent interaction between PDA and rGO interaction seems to affect the HOMO-LUMO level of P/G ink under exposure. Indeed, we have used Gaussian software to examine these complexes at APFD/6-31G* level. The output of the calculation is shown in Table S3, showing that the HOMO-LUMO levels (and band gap in some extent) vary differently for different isomers. This could explain the resistance change of the sensor in the experiment with different xylene isomers; as HOMO-LUMO levels of complexes varied with respect to the Fermi level of the metal electrode of the sensor."*

		In the revised manuscript, we have supplemented more investigation about the mechanism for distinguishing isomers and enantiomer (Supplementary Fig. 25 and 26). The results prove that our chromatography-mimic OHSA design represents an effective and critical mass transport for discriminating structural isomers and enantiomers. We have clarified this aspect in the text by adding (Page 15):<br><br>*"In order to illustrate the mechanism of OHSA for distinguishing VOCs' isomers and enantiomers, one P/G ink-loaded quartz crystal microbalance (QCM) sensor was equipped (Supplementary Fig. 25a and 25b) and three P/G@paper sensors were configured with different layers of paper from 0 to 3 as outside covers (Supplementary Fig. 26a). The P/G ink-loaded QCM sensor was exposed to Xylene isomers and 2-Octanol enantiomers (Supplementary Fig. 25c), and the results demonstrate the absorption or diffusion of such VOC gas molecules onto the film of P/G ink to result in its mass changes but there is negligible mass discrimination between VOC isomers and enantiomers. Three P/G@paper sensors were also challenged with Xylene isomers (Supplementary Fig. 26b-d) and 2-Octanol enantiomers (Supplementary Fig. 25f-h). The P/G@paper sensor without outside cover showed a bit differentiable $(R_{end}-R_b)/R_b$ responses between Xylene isomers (Supplementary Fig. 26b and e). While the P/G@paper sensors covered with 1 or 3 layers of paper can display more distinguishable and layer-dependent $(R_{end}-R_b)/R_b$ responses between Xylene isomers (Supplementary Fig. 26c-e). Additionally, the P/G@paper sensors covered with outside layers can present chiral discrimination toward 2-Octanol enantiomers as the number of layers increases (Supplementary Fig. 26g-i). These results prove that our OHSA design represents a key and effective mass transport for discriminating structural isomers and enantiomers through its unique TSR-HDPR features."*
6.	Is the P/G ink painted directly on skin and fingernail in the supporting video?	Yes. As shown in supporting video and Fig. 1i, the P/G ink painted directly on skin and

	If so, please comment on the safety of the ink to human health. Why is the P/G ink waterproof? Since the P/G ink is dispersed in ethanol, the dried ink seems to be also soluble in water. If the P/G ink is not soluble in water, how can we remove the ink from skin?	fingernail using a vial-based DIY pen loaded with P/G ink (Fig. 1a).<br><br>In the revised manuscript, we performed a cytotoxicity test and animal blood tests for nanotoxicity of P/G ink to verify its biocompatibility (Supplementary Fig. 7). The results of the cytotoxicity test and animal blood tests for nanotoxicity were clarified this aspect in the text by adding (Page 5):<br><br>*"The waterproof of P/G ink dried on skin and fingernail can be attributed to the strong affinity of mussel-inspired PDA matrix with the rGO[23,24]. Removal of the P/G ink from skin and fingernail can be achieved by ethanol. Concerns regarding the safety of the P/G ink were examined via cytotoxicity and blood tests. Blood samples having 293 cell viability examined with WST-8 assay were exposed to 10-100 μg/mL of PDA@rGO for 24 h (Supplementary Fig. 7a). The cell viability showed 102.7±3.1% cell survival even upon exposure to 100 μg/mL concentration of PDA@rGO. The time-course level of immunoglobulin E (IgE, one of immune system markers) in the blood of young Sprague-Dawley rats showed no changes (or changes within the experimental error) when part of their (shaved) skin was covered with P/G ink (Supplementary Fig. 7b). These results indicate that the P/G ink is biocompatible and safe for direct use of animal or human skin"*<br><br>As mentioned in Page 3 of the manuscript, one component of the ink is polydopamine (PDA) which "is a unique catechol-derived molecule mimicking mussel adhesive protein". In the revised manuscript, we discussed the waterproof of P/G ink. And we have clarified this aspect in the text by adding (Page 5):<br><br>*"The waterproof of P/G ink dried on skin and fingernail can be attributed to the strong affinity of mussel-inspired PDA matrix[23,24], which can conjugate with rGO to form a film for coating on skin and fingernail."*<br><br>Because of the dispersibility of P/G ink in ethanol, we can drop some ethanol to remove

		the P/G ink from skin and fingernail. We have clarified this aspect in the text by adding (Page 5):<br><br>*"Because of the dispersibility of P/G ink in ethanol, we can drop some ethanol to remove the P/G ink from skin and fingernail."*

REVIEWER 2		
	**Remarks to the Author**	**Response to the Reviewer**
1.	The idea of applying origami concept to bring a 3D structure is novel….. The use of PCA method to discriminate pure chemical mixtures is not novel and does not have demonstrated practical applications in any current commercial device for the intended purpose reported in this manuscript.<br><br>Furthermore, the sensor array reports changes to multiple chemical and physical stimuli such as temperature, humidity, and pressure. Controlling all these physical variables together with the chemical variables in a real sensing scenario is difficult.	Indeed, as the reviewer noticed, the origami-based structure and implementation is novel and can bring unique added-values for both physical and chemical sensing. The PCA is not claimed to be a novelty of this ms. Rather, it's a representative way for presentation of the results.<br><br>Discrimination between various stimuli in complex environment is indeed difficult, but not impossible. This could be achieved by using array of sensors with wide spectrum of functional groups and by adding calibration curves for each single stimulus to the pattern recognition algorithms can make this happen. As a response to this comment below while providing a proof-of-concept on this matter. Further efforts in these endeavors will be the item of our upcoming researches in this field.
2.	The features of the system could be subjected to the way the sensors are packed, and reproducibility may be an issue if the space between layers is different.	In the revised manuscript, we have investigated the effect of packing on sensors' features and their reproducibility, by parallelly fabricating 21 one-sided OHSA in the same way and assessing the reproducibility of their features of layer-involved resistance changes. The results showed that there is robust reproducibility regarding their features of layer-involved resistance changes. We have clarified this aspect in the text by adding (Page 7):<br><br>*"Considering that the features of the OHSA system may be subjected to the way it is packed (e.g. the space between layers might be*

		different), we further fabricated 21 one-sided OHSA in the same way and assessed the reproducibility of their features, the results showed that there is robust reproducibility regarding their features of layer-involved resistance changes (i.e. RSD values of (R_{end}-R_b)/R_b are all lower than 12%) (Supplementary Fig. 11)."
3.	Mass transport of the processes leading to a response should be discussed. Passive diffusion vs. different convective conditions may offer differences in the response discrimination patterns.	In the revised manuscript, we have performed detailed theoretical modeling, via Prof. Simon Brandon (now, coauthor on the revised manuscript), to investigate the mass transport of the processes leading to the OHSA responses. Detailed description of this analysis could be seen in Page 9-10. Analysis and plots are presented in Supplementary Fig. 13 are qualitatively similar to the above experimental observations in Fig. 2g and 2h. We have clarified this aspect in the text by adding (Page 9-10):<br><br>*"Theoretical modeling was also performed to investigate the mass transport through OHSA. We treated the sensor array as a porous block of material which is initially in vacuum (within a chamber) and is suddenly exposed to a step-rise in pressure. Due to the abrupt rise in pressure it is reasonable to assume that the chamber and the porous material are immediately filled with the sample gas. For simplicity, we assumed the gas contains a single impurity of which the small amount initially entering the porous material is mostly and immediately adsorbed. Thus, at "time=0" the gas phase in the chamber contains a background concentration of the impurity and the gas phase within the pores contains very little impurity which is at equilibrium with a small amount adsorbed onto the porous medium; the system is at uniform pressure. In this case we can treat the problem as a diffusion-adsorption problem described by the following non-dimensional partial differential equation (PDE), initial and boundary conditions:*

$$\frac{\partial c}{\partial t} = \frac{\partial^2 c}{\partial x^2} \qquad t > 0, \quad 0 \le x \le 1$$

$$c = 0 \qquad t = 0, \quad 0 \le x \le 1$$

$$c(t,0) = H(t) \qquad t > 0, \quad x = 0$$

$$\frac{\partial c}{\partial x} = 0 \qquad t > 0, \quad x = 1$$

Here, the dimensionless concentration of impurity is defined as $c \equiv \left(\tilde{c} - \tilde{c}^*\right) / \tilde{c}_0$ *, where "* $\tilde{c}$ *" is the dimensional impurity concentration in the gas phase, "* $\tilde{c}^*$ *" is the small level of dimensional impurity concentration initially in the pores after the first quick adsorption (immediately following the sharp rise in pressure), and "* $\tilde{c}_0$ *" is the background impurity concentration in the chamber (assumed constant - i.e. in the model the chamber is assumed to be infinitely large compared to the porous medium). The dimensionless time is normalized using a characteristic time given by:* $t_c = \left[1 + \frac{(1-\varepsilon)}{\varepsilon} a\right] \frac{L^2}{\left(D\varepsilon / \tau^2\right)}$ *, where "ε" is the porosity, "L" is the thickness of the porous block of material in the one sided case and half the thickness in the two-sided case, "D" is the gas phase diffusion coefficient of the impurity, "τ" is the tortuosity of the porous medium and "a" (for simplicity assumed constant) is the derivative of the adsorption isotherm (concentration of adsorbed material) with respect to the gas phase concentration - i.e.* $a = dq / d\tilde{c}$ *where "q" is the dimensional concentration of adsorbed material (assumed at equilibrium with the gas phase in the pores). Finally, the dimensionless distance "x" is normalized with L and H(t) is the Heaviside step function. The above PDE, initial condition and boundary conditions has an analytical solution given by Carslaw et al[29]:*

$$c(t,x) = \frac{4}{\pi} \sum_{n=0}^{\infty} \frac{(-1)^n}{(2n+1)} \cos\left[\left(\frac{2n+1}{2}\right)\pi(1-x)\right] \left\{1 - Exp\left[-\left(\frac{2n+1}{2}\right)^2 \pi^2 t\right]\right\}$$

Plots of this function for the six (dimensionless) sensor positions within OHSA (porous block of material) are provided below for the one-sided and two-sided cases. Notice that in the two-sided case, due to symmetry,

		only half the system is considered and hence the last two sensors are, in a sense, at the same positions as sensors two-and-three (Supplementary Fig. 13). Finally, these plots in Supplementary Fig. 13 are qualitatively similar to the above experimental observations in Fig. 2g and 2h."
4.	On the other hand, the comparison of time-space resolve chromatography-mimic is misleading because there is no discrimination of different chemicals based on chemical interactions that accelerate or promote the mass transport in a structure to induce responses capture at different times. The term: "time-space-resolved chromatography-mimic OHSA" should be replaced by a name reflecting the actual system, example: "high discrimination PCA OHSA"	The use of PCA shall be considered as a representative approach for presentative the results. Therefore, it would be less relevant to include it in the term describing the reported device. However, inspired by your comment, we have now changed the term "time-space-resolved chromatography-mimic" to "**time-space-resolved high-discriminative pattern recognition** (TSR-HDPR)".
5.	There are several exaggerative sentences that should be completely eliminated or replaced given the system does no necessary applies to the specific mentioned applications. Examples are:<br><br>*Therefore, our time-space-resolved chromatography-mimic OHSA could reach help reach a future goal for non-invasive medical diagnostics based on the analysis of VOCs emitted from "diverse bodily sources".<br><br>*It should provide a potential tool for profiling and deciphering homologous VOCs (e.g. enantiomers) toward "precision medicine".<br><br>*Because of these intriguing features, the OHSA-based hierarchical design presented herein opens the door for the creation of smart time-space-resolved chromatography-mimic electronics, and this would boost more versatile and meaningful intelli-sense applications toward precise medicine, healthcare monitoring, etc.	In the revised manuscript, we have eliminated these sentences.

REVIEWER 3		
	**Remarks to the Author**	**Response to the Reviewer**
**General comments:**		
	The manuscript describes the low-cost fabrication and properties of PDA-rGO based origami sensor array for the detection of different physical (temperature, humidity, pressure, light) and chemical (VOC) stimuli. One of the uniqueness of this sensor is its ability to identify structural isomers and chiral recognition of enantiomers. Although the scientific content is exciting to read, the grammar and English should be improved considerably.	We thank for the reviewer's comments and appreciate the reviewer's feedback, which has helped us make significant improvement of the quality of this manuscript.
**Specific comments:**		
1.	In section 'P/G ink-loaded origami hierarchical sensor array (OHSA)' authors should consider identifying the type of paper used for the origami sensor and the thickness/amount of the ink deposited on the substrate. It would improve the manuscript if the authors commented on the effect of thickness of ink on the adhesion on various substrates? Do paper type and paper quality affect the resistivity readings?	In the revised manuscript, we have further investigated the effect of paper's type, paper's quality and ink amount on the resistivity reading (Fig. 5e and f, Supplementary Fig. 28). From the results, the integration of OHSA-based hierarchical design and chemical engineered inks or diversified paper substrates or various amounts of ink used can tune the resistivity reading for potentially spawning more TSR-HDPR electronics toward widespread applications. We have clarified this aspect in the text by adding (Page 17):<br><br>*"In addition, the nature of paper is such that the bonding of its fibers induces many tiny air passages throughout it (i.e. porosity). Paper porosity affects how thoroughly and how quickly inks are absorbed into a paper primarily by capillary action, and the length of time it takes for air to permeate/diffuse a paper or the rate of the passage of air through it. Thus, another potential way to tune the configuration of origami for regulating its TSR-HDPR characteristics can be adopted by using porous papers with different qualities. In response to this, another three one-sided OHSAs have been fabricated by using three types of paper (thickness in Thin, Middle and Thick with different porosity, Fig. 5e) as substrates written with the same ink, and such OHSAs were also exposed to vacuum and air*

		in the same way as the test in Fig. 2h. Fig. 5f describes that various thicknesses of papers as substrates can also modulate the layer-related characteristics of relative resistance changes to air exposure, thinner paper gives rise to more extensive responses. We also examined the effect of ink amount deposited on the substrate toward the resistivity readings of OHSA (Supplementary Fig. 28), manifesting that varied amounts of ink used can change the thickness of ink film for likewise regulating the layer-related characteristics of relative resistance changes to air exposure."
2.	What is the meaning of 'vacuum clean'? Was there a sub-atmospheric pressure in a stainless-steel chamber to remove 'air species'? As per figure 8a and b in the supplemental materials, the pressure in the chamber was varied from 793 to 868 Torr- these pressures are not associated with the generally accepted definition of the term vacuum.	The "vacuum clean" means that the exposure chamber is under the pressure of 0.06 Torr read by a pressure meter, which uses to remove the air species absorbed on the P/G ink films. We have clarified this aspect in the text by adding (Page 8):<br><br>*"The "vacuum clean" means that the exposure chamber is under the pressure of 0.06 Torr read by a pressure meter, which uses to remove the air species absorbed on the P/G ink films."*<br><br>And in Supplementary Fig. 8c, 19a and 19b, the pressure from 793 to 868 Torr in the chamber is air pressure we tested with our sensor. It is not under vacuum.
3.	Figure 2g and h: the relative resistivity seems to saturate with time. Did authors observe this saturation? If yes, what are the saturation values for different layers?	Yes, the relative resistivity can saturate with time, and the saturation values for different layers may be corrected with the OHSA's features of layer-related resistance changes within different air exposure time. Therefore, in the revised manuscript, we further evaluated the layer-relevant resistance changes of one-sided OHSA challenged with different air exposure time (e.g., 10 min and 45 min) (Supplementary Fig. 10), and the results revealed that different exposure time has little effect on the OHSA's features of layer-related resistance changes. We have clarified these points in the text by adding (Page 7):<br><br>*"Since the air-induced relative resistivity of OHSA was noticed to saturate with time (Fig. 2g and h), we then evaluated the layer-relevant*

		resistance changes of one-sided OHSA challenged with different air exposure time (Supplementary Fig. 10), and the results revealed that different exposure time has little effect on the OHSA's features of layer-related resistance changes."
4.	In section 'Multifunctional monitoring of multiple stimuli by using OHSA' authors state: Notably from Supplementary Fig. 12b and d, it can be seen that most of the tested VOCs were clearly differentiated in the PCA plots using both two-sided OHSA and one-sided OHSA. However, in figure 3i and j and in figure 12 b and d, values for acetic acid, heptane, nonane etc. do overlap. Authors need to address this.	Yes, both the two OHSAs can discriminate each stimulus (e.g., VOCs) with different levels by PCA, and from Supplementary Fig. 17b and d (in the revised manuscript), most of the tested VOCs can be clearly differentiated in the 2D PCA plot. When we took all the stimuli (including temperature, RH, light, and VOCs) into account via the same PCA, the canonical resistance-response patterns from all the above stimuli can be also fully classified into the independent area of 2D PCA plot (Fig. 3i and j), but some of the tested VOCs were noticed to overlap due to the comprehensive extraction of features from all the above stimuli, and the degree of overlap in the case of two-sided OHSA is more significant than that of one-sided OHSA (Fig. 3i and j). This phenomenon can be also attributed to the symmetry of two-sided OHSA for bidirectional stimuli permeation/diffusion. As for addressing the overlap issue, we used 3D PCA plots to differentiate the canonical resistance-response patterns from VOCs (Supplementary Fig. 20). We have clarified this aspect in the text by adding (Page 12-13):<br><br>*"Notice that there are some overlap between the canonical resistance-response patterns from the tested VOCs in the 2D PCA plots using both the two OHSAs, we further employed 3D PCA plots to differentiate the canonical resistance-response patterns from VOCs (Supplementary Fig. 20). As a result, most of the tested VOCs (including acids, alkanes, ketones, aldehydes and alcohols, listed in Table S1) were differentiated using two-sided OHSA (Supplementary Fig. 20a) and all the tested VOCs were well distinguished using one-sided OHSA (Supplementary Fig. 20b), suggesting that one-sided OHSA would have stronger discrimination toward VOCs due to its unique*

		one entrance for the random permeation/diffusion of VOCs."
5.	Since both one sided and two-sided origami arrays can be used to detect the physical and chemical stimuli, authors should discuss advantages of using one sided and two sided OHSA over each other.	There was no obvious difference in our test settings between two-sided OHSA and one-sided OHSA in response to temperature (Fig. 3a and e) and light exposure (Fig. 3c and g) because of their unidirectional transmission; that is, the resistance responses of these two OHSA's layers had monodirectional gradient tendencies toward temperature (Supplementary Fig. 14) and light exposure (Supplementary Fig. 16). Regarding the monitoring of RH and VOCs, two-sided OHSA presented nearly symmetrical resistance responses toward these stimuli from the outer layers to the inner layers (Fig. 3b and d, Supplementary Fig. 15a-b and 17a); by contrast, one-sided OHSA clearly showed one-way responses (Fig. 3f and h, Supplementary Fig. 15e-f and 17b). These diverse results can be attributed to the random permeation/diffusion of RH and VOCs into the two entrances of two-sided OHSA and one entrance of one-sided OHSA (Fig. 2d-2f). Moreover, most of the tested VOCs (including acids, alkanes, ketones, aldehydes and alcohols, listed in Table S1) were differentiated using two-sided OHSA (Supplementary Fig. 20a) and all the tested VOCs were well distinguished using one-sided OHSA (Supplementary Fig. 20b), suggesting that one-sided OHSA would have stronger discrimination toward VOCs due to its unique one entrance for the random permeation/diffusion of VOCs. We have clarified this aspect in the text in the revised manuscript, by adding (Page 12-13):<br><br>*"As a result, most of the tested VOCs (including acids, alkanes, ketones, aldehydes and alcohols, listed in Table S1) were differentiated using two-sided OHSA (Supplementary Fig. 20a) and all the tested VOCs were well distinguished using one-sided OHSA (Supplementary Fig. 20b), suggesting that one-sided OHSA would have stronger discrimination toward VOCs due to its unique one entrance for the random permeation/diffusion of VOCs."*

6.	What will be the response of OHSA if instead of zigzag folding, each individual layer is cut at the fold and stacked on top of each other?	Due to the analogous hierarchical structure, the response of layer-by-layer OHSA, which is made of individual layer cut at the fold and stacked on the top of each other, is alike to that of the zigzag folding OHSA (Supplementary Fig. 12). Therefore, the OHSA design can be potentially extended to other strategies. Compared to the layer-by-layer strategy, zigzag folding would be a more controllable and integrated framework for the encapsulation of OHSA toward further applications. We have clarified this aspect in the text in the revised manuscript by adding (Page 7-8):<br><br>*"Apart from OHSA with symmetrical zigzag folding, we also fabricated OHSA with layer-by-layer strategy, in which individual layer was cut at the fold and stacked on the top of each other. Due to the analogous hierarchical structure, the response of layer-by-layer OHSA is alike to that of the zigzag folding OHSA (Supplementary Fig. 12). Therefore, the OHSA design can be readily extended to other strategies. Compared to the layer-by-layer strategy, zigzag folding would be a more controllable and integrated framework for the fabrication of OHSA toward further applications."*

REVIEWERS' COMMENTS:

Reviewer #1 (Remarks to the Author):

I am almost satisfied with the response to my comments.
Minor comments are shown below.
After the revision, I recommend this manuscript in publication.

1. The unit of S/m is conductivity, not sheet resistivity.
2. Some units (maybe oC) disappear in the caption of Supplementary Fig. 22.
3. Please show how you can fabricate the gold-plated QCM resonator in Figure S25. Just coating the P/G ink on a commercial QCM resonator ?

Shinya Kano, Kobe University

Reviewer #2 (Remarks to the Author):

The authors have addressed most of the concern points. However, one of the main points for discussion remains to be clarified in the text of the manuscript despite the fact the authors acknowledge it in the reply letter.
The point is related to the usability of the presented sensor system in "real sensing scenarios", meaning with real scenarios where temperature, humidity, pressure, and chemicals can change any time.
To address the specific concern, this reviewer suggest to add in the discussion portion the following statement, which is a statement modified from the author's reply.

"Discrimination between various physical and chemical stimuli in complex changing environments is difficult to achieve with a sensor array based solely in chemical interactions. However, a pre-calibrated array of chemical and physical sensors would allow to achieve the goal. Sensors immune to chemical analytes, and solely dedicated for measuring physical parameters such as temperature, humidity, barometric pressure could be used to assess the physical conditions, and enhance the features of the here presented system, further improving chemical discrimination capabilities in such complex and changing environments."

Actions (revisions) appear in red; in the marked-up revised version of the manuscript. Those changes are highlighted. Text from the original or revised manuscript appears in *italics*.

REVIEWER 1		
	**Remarks to the Author**	**Response to the Reviewer**
**General comments:**		
	I am almost satisfied with the response to my comments. Minor comments are shown below. After the revision, I recommend this manuscript in publication.	We thank for your positive comments and appreciate your feedback. In the revised manuscript, we have carefully addressed your concerns and improved the quality of our work.
**Specific comments:**		
1.	The unit of S/m is conductivity, not sheet resistivity.	In the revised manuscript, we recalculated the sheet resistivity with correct unit. Also, we have clarified this aspect in the text by adding (Page 4):<br><br>*"its sheet resistance is ~5.479×10^{-4} Ω•m"*
2.	Some units (maybe ^{o}C) disappear in the caption of Supplementary Fig. 22.	We have refined the caption of Supplementary Figure 22.
3.	Please show how you can fabricate the gold-plated QCM resonator in Figure S25. Just coating the P/G ink on a commercial QCM resonator?	Indeed, we have just coated the P/G ink on a commercial QCM resonator. In the revised manuscript, we have clarified this aspect in the text (Page 14) and the caption of Supplementary Figure 25 by adding:<br><br>*"P/G ink-coated"*

REVIEWER 2		
	**Remarks to the Author**	**Response to the Reviewer**
	The authors have addressed most of the concern points. However, one of the main points for discussion remains to be clarified in the text of the manuscript despite the fact the authors acknowledge it in the reply letter. The point is related to the usability of the presented sensor system in "real sensing scenarios", meaning with real scenarios where temperature, humidity, pressure, and chemicals can change any time.	We have now supplemented the modified statement according to the reviewer's suggestion by adding (Page 12):<br><br>*"discrimination between various physical and chemical stimuli in complex changing environments is difficult to achieve with a sensor array based solely in chemical interactions. However, a pre-calibrated array of chemical and physical sensors would allow to achieve the goal. Sensors immune to chemical analytes, and solely dedicated for*

	To address the specific concern, this reviewer suggests to add in the discussion portion the following statement, which is a statement modified from the author's reply.<br><br>"Discrimination between various physical and chemical stimuli in complex changing environments is difficult to achieve with a sensor array based solely in chemical interactions. However, a pre-calibrated array of chemical and physical sensors would allow to achieve the goal. Sensors immune to chemical analytes, and solely dedicated for measuring physical parameters such as temperature, humidity, barometric pressure could be used to assess the physical conditions, and enhance the features of the here presented system, further improving chemical discrimination capabilities in such complex and changing environments."	*measuring physical parameters (such as temperature, RH) could be used to assess the physical conditions, and enhance the features of the here presented system, further improving chemical discrimination capabilities in such complex and changing environments."*
